# The PBAF chromatin remodeling complex contributes to metal homeostasis through MTF1 regulation

**DOI:** 10.1093/mtomcs/mfag019

**Published:** 2026-06-05

**Authors:** Nick Carulli, Emma E Johnston, David C Klein, Odette Verdejo-Torres, Anand Parikh, Arianna McDaniels, Antonio Rivera, Michael Quinteros, Aidan T Pezacki, Christopher J Chang, Sarah J Hainer, Teresita Padilla-Benavides

**Affiliations:** Department of Molecular Biology and Biochemistry, Wesleyan University, Middletown, CT 06459, United States; Department of Molecular Biology and Biochemistry, Wesleyan University, Middletown, CT 06459, United States; Department of Biological Sciences. University of Pittsburgh, Pittsburgh, PA 15260, United States; Department of Molecular Biology and Biochemistry, Wesleyan University, Middletown, CT 06459, United States; Department of Molecular Biology and Biochemistry, Wesleyan University, Middletown, CT 06459, United States; Department of Molecular Biology and Biochemistry, Wesleyan University, Middletown, CT 06459, United States; Department of Molecular Biology and Biochemistry, Wesleyan University, Middletown, CT 06459, United States; Department of Molecular Biology and Biochemistry, Wesleyan University, Middletown, CT 06459, United States; Department of Chemistry, Princeton University, Princeton, NJ 08544, United States; Department of Chemistry, University of California, Berkeley, CA 94720, United States; Department of Chemistry, Princeton University, Princeton, NJ 08544, United States; Department of Chemistry, University of California, Berkeley, CA 94720, United States; Department of Molecular and Cell Biology, University of California, Berkeley, CA 94720, United States; Department of Biological Sciences. University of Pittsburgh, Pittsburgh, PA 15260, United States; Department of Molecular Biology and Biochemistry, Wesleyan University, Middletown, CT 06459, United States

## Abstract

Chromatin remodeling by SWI/SNF complexes is essential for transcriptional regulation, yet how distinct SWI/SNF subcomplexes contribute to cellular stress responses remains incompletely understood. Here, we identify a specific role for the PBAF subunit Baf180 in regulating metal-responsive transcription and adaptation to metal stress in proliferating myoblasts. While knockdown (KD) of the BAF-specific subunit *Baf250a* or the ncBAF-specific subunit *Brd9* significantly impairs myoblast proliferation, KD of the PBAF-specific subunit *Baf180* has no effect under basal conditions. Notably, supplementation with copper (Cu) or zinc (Zn) restores proliferative capacity in *Baf250a*- and *Brd9*-deficient myoblasts. In contrast, *Baf180*-depleted myoblasts exhibit impaired proliferation upon metal exposure, accompanied by selective dysregulation of genes involved in Cu and Zn homeostasis. Transcriptomic and chromatin profiling further reveal that loss of Baf180 alters the activity of metal-regulatory transcription factor 1 (MTF1), including reduced chromatin occupancy at metal-responsive loci. Together, these findings support a model in which PBAF promotes metal-responsive gene regulation to maintain metal homeostasis and sustain myoblast proliferation, uncovering a previously unrecognized link between nucleosome remodeling and metal homeostasis during muscle cell proliferation.

## Graphical Abstract

**Graphical Abstract:**
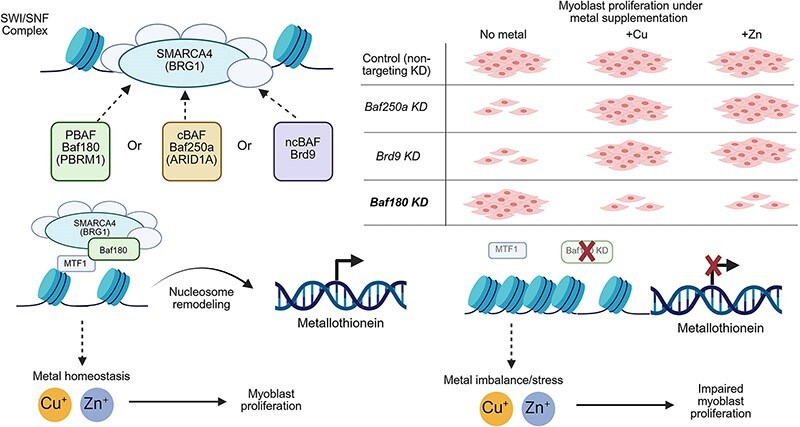

## Introduction

In mammalian cells, genomic DNA is packaged into chromatin, a hierarchical structure in which ∼150 bp of DNA is wrapped around histone proteins to form nucleosomes, limiting access of transcription factors to the genome [1, 2]. Chromatin accessibility is dynamically regulated through post-translational histone modifications and ATP-dependent nucleosome remodeling, which together control gene expression programs essential for development and cell identity [3–7]. SWI/SNF chromatin remodeling complexes use the energy of ATP hydrolysis to reposition or evict nucleosomes, thereby facilitating transitions between repressive and permissive chromatin states [6, 7]. All mammalian SWI/SNF complexes contain one of two mutually exclusive ATPase subunits, Brahma (BRM) or Brahma-related gene 1 (Brg1) and are further divided into three major subcomplexes: canonical BAF (cBAF), polybromo-associated BAF (PBAF), and non-canonical BAF (ncBAF), distinguished by their unique subunit compositions [8–11]. cBAF complexes contain subunits such as Baf250a and Dpf2, PBAF complexes are defined by Baf180, Baf200, and Brd7, and ncBAF complexes contain Brd9 and GLTSCR1 [11–14]. Although these complexes share a common remodeling core, growing evidence indicates that they perform specialized and context-dependent functions across tissues and developmental stages.

Consistent with this idea, individual SWI/SNF subunits have been shown to regulate distinct transcriptional programs in diverse biological settings. For example, cBAF components such as BRG1 and Baf250a are required for glucocorticoid receptor (GR)-mediated chromatin remodeling and hormone-responsive gene expression, whereas PBAF subunit Baf180 is dispensable in this context [15–17]. Similarly, Baf60C plays critical roles in insulin-responsive transcription in the liver and in cardiac and skeletal muscle development, whereas its depletion affects myogenic differentiation [18, 19]. Baf250a is also essential for cardiac progenitor development, with its depletion impairing cardiomyocyte differentiation through misregulation of key transcription factors [20]. In contrast, ncBAF has emerged as a regulator in cancer cells, where Brd9 interacts with nuclear hormone receptors and supports cell proliferation and survival [21, 22].

The PBAF subcomplex has been implicated in additional cellular processes, including cell cycle regulation, genome stability, and nuclear-receptor mediated transcription. Loss of Baf180 leads to premature senescence, disrupted hematopoiesis and aberrant cell cycle control, in part through derepression of p21 [23, 24]. PBAF also plays a role in maintaining genome integrity by suppressing transcription near DNA double-strand breaks (DSBs) and facilitating DNA repair in an ATM kinase-dependent manner [25]. In developmental contexts, Baf180 is required for proper cardiac and placental development, phenocopying defects observed upon loss of nuclear receptors such as retinoic acid receptor-α (*RXRα*), vitamin D receptor (*VDR*), and peroxisome proliferator-activated receptor γ (*PPARγ*) [26]. Together, these studies highlight the diverse and context-specific functions of PBAF, while highlighting that its roles in many biological processes remain incompletely understood.

Our group has previously examined the role of cBAF, ncBAF, and PBAF complexes during skeletal muscle lineage progression [12, 13, 27]. Using subunit-specific knockdown (KD) of *Baf250a* (cBAF), *Brd9* (ncBAF), and *Baf180* (PBAF) in C2C12 myoblasts, we demonstrated that the cBAF is essential for myoblast proliferation and differentiation through regulation of myogenic transcription factors, including *Pax7* and *Myogenin* [12, 13]. In contrast, ncBAF contributes indirectly to myogenesis through effects on RNA metabolism, while PBAF appeared largely dispensable for myogenic gene expression and differentiation [12, 13, 28]. However, depletion of *Baf180* resulted in impaired expression of stress-related genes, including genes involved in metal transport and homeostasis [12], suggesting a novel function for PBAF in this context.

Metal ions such as copper (Cu) and zinc (Zn) are essential for skeletal muscle development, supporting mitochondrial respiration, enzymatic activities, and redox balance. Cu is specifically required for myoblast proliferation and differentiation, and disruption of Cu homeostasis impairs myogenesis [29, 30]. Metal-responsive transcription regulation in muscle is mediated in part by metal regulatory transcription factor 1 (MTF1), a Zn-finger transcription factor that responds to intracellular metal availability [31] and is required for myoblast differentiation [32]. In myoblasts, MTF1 regulates expression of metal transporters such as PiC2, which is upregulated during myogenesis and necessary for proper proliferation and differentiation, as well as additional factors involved in maintaining metal homeostasis, including Crip2 [32–34]. Despite the importance of both nucleosome remodeling and metal-responsive transcription in muscle biology, the intersection of these pathways remains poorly defined.

In this study, we investigate the role of SWI/SNF subcomplexes in metal-responsive gene regulation during myogenesis. We hypothesized that PBAF contributes to myoblast proliferation by modulating metal-responsive gene networks, potentially through interactions with MTF1. Using shRNA-mediated KD of cBAF, PBAF, and ncBAF subunits in C2C12 myoblasts, we found that Zn or Cu supplementation differentially affects proliferation depending on SWI/SNF subcomplex integrity, with *Baf180*-deficient cells exhibiting a unique sensitivity to metal stress. Collectively, these data define a link between nucleosome remodeling, metal homeostasis, and myoblast proliferation.

## Materials and methods

### Antibodies

Primary antibodies used include hybridoma supernatant against Pax7 (Developmental Studies Hybridoma Bank, University of Iowa; deposited by A. Kawakami), for immunocytochemistry. Mouse anti-MTF1 (sc-365090, Santa Cruz Biotechnology), rabbit anti-Baf180, rabbit anti-Baf250a, anti-GAPDH (A0334, A16648, A19056, respectively) were from Abclonal. The rabbit anti-Brd9 (PA5-113488) was from Thermo Fisher Scientific. For confocal microscopy, rabbit anti-Baf180 (89123S), anti-Baf250a (1235S), and anti-Brd9 (48306S) were from Cell Signaling Technology were used at 1:50, 1:50, and 1:100 dilutions, respectively. Secondary antibodies included HRP-conjugated anti-rabbit IgG (31 460), goat anti-rabbit IgG Alexa Fluor Plus 594, goat anti-mouse IgG Alexa Fluor Plus 488, goat anti-rabbit IgG Alexa Fluor Plus 633, and donkey anti-mouse Alexa Fluor 594 (A32740, A32723, A21070, and A32744 were from Thermo Fisher Scientific). All antibodies were validated for specificity and used at the indicated dilutions.

### C2C12 myoblast culture

Immortalized murine myoblast C2C12 and HEK293T cells were purchased from American Type Culture Collection (ATCC) and cultured in proliferation media composed of Dulbecco’s modified Eagle’s medium (DMEM) supplemented with 10% fetal bovine serum (FBS) and 1% penicillin–streptomycin. Cells were maintained in a humidified incubator at 37°C with 5% CO_2_ at subconfluent densities.

### Virus production, and transduction of C2C12 myoblasts

Mission plasmids encoding shRNAs targeting specific subunits of mSWI/SNF complexes were obtained from Sigma and used as previously reported [12, 13]. *Baf250a* was selected for the BAF complex, and *Baf180* and *Brd9* for the PBAF and ncBAF, respectively (Supp. Table 1). The shRNA constructs (15 µg), along with packaging vectors pLP1 (15 µg), pLP2 (6 µg), and pSVGV (3 µg), were transfected into HEK293T cells using lipofectamine 2000 (Thermo Fisher Scientific) following the manufacturer’s instructions. The next day, the culture medium was replaced with 10 ml fresh DMEM enriched with 10% FBS. The viral supernatant was collected at 24 and 48 h and passed through a 0.22 µm syringe filter (Millipore). For myoblast infection, 5 ml of the filtered supernatant, supplemented with 8 μg/ml polybrene (Sigma), were used to transduce 2 × 10^6^ cells, following established protocols [12, 13]. Following an overnight incubation, the infected cells underwent selection in growth media containing 2 μg/ml puromycin (Invitrogen). The stable myoblast population was then sustained in growth media supplemented with 1 μg/ml puromycin.

### Western blot analyses

C2C12 myoblasts were washed with PBS and solubilized with RIPA buffer (10 mM Tris pH 8, 1% Triton X-100, 0.1% sodium deoxycholate, 0.1% sodium dodecyl sulfate (SDS), 140 mM sodium chloride) and 100 µl of Complete protease inhibitor cocktail (PIC; Thermo Scientific). Lysates were sonicated for 12 cycles, and protein was quantified via Bradford assay [35]. Samples (20 µg) were separated on SDS-PAGE gels and transferred to PVDF membranes (Millipore). Proteins were detected with primary and HRP-conjugated secondary antibodies and visualized with chemiluminescence (Tanon, Abclonal Technologies). Band intensity was quantified using ImageJ and normalized to GAPDH.

### Cell proliferation assays

C2C12 myoblasts were initially seeded at a density of 1 × 10^4^ cells/cm^2^ in the presence of increasing concentrations of CuSO_4_ (50, 100, 200, 300, and 500 µM) and ZnSO_4_ (50, 100, and 150 µM). Samples were trypsinized at 24, 48, and 72 h post-seeding and cell count and viability were determined using a Spectrum Cellometer from Nexcelcom Biosciences.

### Immunocytochemistry and light microscopy

C2C12 myoblasts were grown for 48 h in the presence and absence of increasing concentrations of CuSO_4_ (50, 100, 200, and 300 µM) and ZnSO_4_ (50, 100, and 200 µM). Cells were then fixed overnight at 4°C in 10% formalin in phosphate buffered saline (PBS), washed three times with PBS and permeabilized with Triton X-100 for 10 min. Hybridoma supernatant against Pax7 was used for immunocytochemistry and developed with the universal ABC kit (Vector Laboratories, PK-6200). Images were acquired with an Echo Rebel (Discover Echo) microscope using the 20x objective.

### Immunofluorescence and confocal microscopy

Proliferating C2C12 myoblasts were seeded at 1 × 10^4^ cells/cm^2^ on coverslips with or without supplementation of 100 µM CuSO_4_ and 50 µM ZnSO_4_ for 48 h. Then, the cells were fixed with 10% formalin in PBS, permeabilized with PBT buffer containing 0.5% Triton X-100 in PBS, and blocked in 5% horse serum in PBT for 1 h. Next, the samples were incubated with the primary antibodies diluted in blocking solution overnight at 4°C. The following day, the myoblasts were washed three times with PBT for 10 min at room temperature and incubated with the corresponding secondary fluorescent antibodies in blocking solution for 2 h at room temperature. Nuclei were stained for 30 min with DAPI. Samples were mounted with VectaShield solution (Vector Laboratories) and imaged with a Leica SP8 using the 63X water immersion lens. Images were analyzed with the Leica Application Suite X (Leica Microsystem Inc). Colocalization was quantified via adjusting the Color Threshold of the image and analyzing the particles using Fiji ImageJ software (Version 4.14.0/1.54f) [36].

### CUT&RUN

CUT&RUN was performed as previously described [34, 37–41], using recombinant Protein A-MNase (pA-MNase) of two independent biological samples [42]. Briefly, nuclear extraction was performed on 100 000 cells with a hypotonic buffer (20 mM HEPES-KOH, pH 7.9, 10 mM KCl, 0.5 mM spermidine, 0.1% Triton X-100, 20% glycerol and protease inhibitors) and bound to lectin-coated concanavalin A magnetic beads (40 µl beads per 100 000 nuclei; Polysciences). Bead-bound nuclei were chelated with blocking buffer (20 mM HEPES, pH 7.5, 150 mM NaCl, 0.5 mM spermidine, 0.1% BSA, 2 mM EDTA and protease inhibitors) and washed (wash buffer: 20 mM HEPES, pH 7.5, 150 mM NaCl, 0.5 mM spermidine, 0.1% BSA and protease inhibitors). Nuclei were incubated for 1 h in wash buffer containing primary antibody (anti-MTF1 (H-6) sc-365090, Santa Cruz Biotechnologies, lot 0 050 480 101; rabbit polyclonal IgG, Abcam ab37415, lot #GR3208186-1), followed by 30-min restriction in pA-MNase diluted in wash buffer, at room temperature with rotation. Samples were equilibrated to 0°C in an ice-water bath and 3 mM CaCl_2_ was added to activate pA-MNase. After 22 min, the digestion reaction was chelated with 20 mM EDTA and 4 mM EGTA, and 1.5 pg MNase-digested *S. cerevisiae* mononucleosomes were added as a spike-in control. Genomic fragments were released after an RNase A treatment and subsequent centrifugation. Isolated fragments were used as input for a library build consisting of end repair and adenylation, ligation of NEBNext stem-loop adapters, and purification with AMPure XP beads (Agencourt). Barcoded fragments were amplified by 15 cycles of high-fidelity PCR and purified using AMPure XP beads. Libraries were pooled and sequenced on an Illumina NextSeq2000 to a depth of ∼10 million uniquely mapped reads.

Paired-end fastq files were trimmed to 25 bp and mapped to the mm10 genome with bowtie2 (options -q -N 1 -X 1000) [43]. Duplicate reads were identified and removed with Picard [44]. Reads were filtered for mapping quality (MAPQ ≥ 10) with SAMtools [45] and size classes corresponding to factor-bound footprints (1-120 bp) were generated using a custom awk script [45]. Reads were converted to BigWig files using deepTools with RPKM normalization (options -bs 1 --normalizeUsing RPKM) [46]. CUT&RUN peaks were called using HOMER, with IgG-targeted CUT&RUNs used as controls, using default parameters [47]. Heatmaps were plotted using deepTools computeMatrix (options -a 2000 -b 2000 -bs 20 --missingDataAsZero) and plotHeatmap [46]. Gene Ontology (GO) analysis was performed on peaks present in both CUT&RUN replicates using Metascape [48]. To control for myoblast-specific background, we kept only genes with a DESeq2 baseMean ≥ 1 (RNA-seq) and used a background list of all genes with a baseMean ≥ 1 in ES cells. *P*-values were corrected for multiple testing via the Benjamini-Hochberg correction (FDR = 0.05).

### Chromatin immunoprecipitation assays

Three independent biological replicates of proliferating primary myoblasts were cross-linked with 1% formaldehyde for 10 min at room temperature on an orbital shaker. Cross-linking was quenched by adding 1 ml of 1 M glycine followed by incubation for 5 min at room temperature with gentle shaking. Cells were then washed three times with 10 ml of ice-cold PBS supplemented with Complete™ Protease Inhibitor (Roche). Cross-linked myoblasts were resuspended in 1 ml of ice-cold PBS containing protease inhibitors and centrifuged at 5000 × *g* for 5 min at 4°C. The supernatant was removed and the cell pellet was resuspended in 200 μl of ice-cold SDS lysis buffer (50 mM Tris–HCl pH 8.0, 10 mM EDTA, 1% SDS) and incubated for 10 min on ice. Chromatin was fragmented by sonication using a Bioruptor UCD-200 (Diagenode). Cells were sonicated three times for 5 min (30 s on/30 s off) at mild intensity. Sonicated samples were immunoprecipitated with the SimpleChIP Plus Enzymatic Chromatin IP Kit (9005, Cell Signaling Technologies), following the manufacturer’s instructions, then the crosslinked myoblasts lysates were incubated for 2 h at 4°C with diluted rabbit anti-MTF1 antibody or rabbit IgG as a control, which were also used for CUT&RUN experiments. Samples were then incubated with 80 μl Magna ChIP Protein A/G Magnetic Beads (MilliporeSigma) and overnight incubation at 4°C with rotation. Bead-bound immune complexes were captured using a magnetic rack and sequentially washed with the corresponding ChIP buffers and immune complexes were eluted in 100 μl of elution buffer. Reverse cross-linking was performed by adding 20 μl of 5 M NaCl and incubating samples overnight at 65°C.

DNA was purified using the ChIP DNA Clean & Concentrator kit (Zymo Research, Irvine, CA, USA) according to the manufacturer’s instructions and stored at -80°C until further analysis by semi-quantitative real-time PCR (qPCR).

### RNA sequencing

RNA sequencing (RNA-seq)  was performed as previously described [41]. Two independent biological replicates of control scramble (scr) and shRNA transduced myoblasts were treated with 100 µM CuSO_4_ and 50 µM ZnSO_4_, or no metal as detailed above. Cell pellets were then flash-frozen in liquid nitrogen. RNA was extracted from frozen pellets with TRIzol per manufacturer’s instructions and purified by chloroform extraction and isopropanol precipitation. Extracted RNA was flash-frozen in liquid nitrogen and stored until use. Ribosomal RNA was removed from 2 µg of input RNA via antisense tiling oligonucleotides and digestion using thermostable RNase H (MCLabs) [49, 50]. rRNA-depleted RNA samples were treated with Turbo DNase (ThermoFisher) and purified by silica column (Zymo RNA Clean & Concentrator kit). rRNA-depleted RNA obtained from each sample was used to build strand-specific RNA-seq libraries using the NEBNext Ultra II Directional Library kit. RNA was fragmented at 94°C for 15 min and used as input for cDNA synthesis and to build strand-specific libraries following the manufacturer’s instructions. Libraries were amplified for 8 cycles of high-fidelity PCR, pooled and sequenced to a depth of approximately 20 million uniquely mapped reads on an Illumina NextSeq2000.

### RNA-seq data analysis

Paired-end fastq files were aligned to the mm10 mouse genome using STAR (options --outSAMtype BAM SortedByCoordinate --outFilterMismatchNoverReadLmax 0.02 --outFilterMultimapNmax 1). RNA-seq data was visualized by generating bigwigs files using deepTools with TPM read normalization (options -bs 5 --smoothLength 20 --normalizeUsing BPM) [46]. Feature counts were generated using subread featureCounts (options -s 2 -p -B) [51]. The count files were imported into R, and subsequent analysis was carried out utilizing DESeq2, implementing the apeglm log_2_ fold change shrinkage correction [52]. GO analysis was performed on significantly up- and downregulated genes using Metascape [48]. The gene lists utilized for GO analysis comprised exclusively significantly DEG with a DESeq2 baseMean value of ≥1, thereby excluding genes with low expression levels. Significance was determined by a DESeq2 adjusted *P*-value < .05.

### Integration of CUT&RUN and RNA-seq datasets

CUT&RUN and RNA-seq datasets were integrated based on gene lists that were called significantly up- or downregulated per DESeq2 results (padj. < .05) and CUT&RUN peak files as described above. A bed file containing differentially expressed gene (DEG) promoters was generated using the UCSC Table Browser [53], with promoters defined as regions spanning 1 kb upstream of UCSC RefGene transcription start sites. Direct overlaps between CUT&RUN peaks and DEG promoters were assigned using the HOMER mergePeaks function (options -d given) [47]. Overlapping regions were annotated using the HOMER annotatePeaks.pl function and the resulting file was manually separated by specific overlap groups [47]. Bed files for each combination of CUT&RUN peaks and DEG promoter regions were generated and used as input for subsequent Gene Ontology, Genome Ontology, and motif enrichment analyses via the HOMER annotatePeaks.pl and findMotifs.pl functions [47]. A background gene list of all genes expressed in these experiments (DESeq baseMean value ≥ 1) was used to control for potential overrepresented motif sequences.

### Immunoprecipitation

Three independent biological replicates of C2C12 myoblasts were seeded at a density of 1 × 10^4^ cells/cm^2^ with or without metal solution and incubated for 48 h. Cells were washed three times with PBS, and scrapped off the plates in lysis buffer (50 mM Tris–HCl, pH 7.5, 150 mM NaCl, 1% Nonidet P40, 0.5% sodium deoxycholate, and Complete Protease Inhibitor Cocktail) supplemented with either anti-MTF1, anti-Baf180 or IgG for 2 h at 4°C, as previously reported [33, 54]. PureProteome protein A/G mix magnetic beads (Millipore) were added to each tube and samples were incubated overnight at 4°C on a shaker. The next day, samples were washed three times with 100 mM NaCl in PBS using a magnetic rack to retain the beads. Immunoprecipitated proteins were then eluted from the beads with freshly prepared elution buffer containing 10% glycerol, 50 mM Tris–HCl pH 6.8, and 1 M NaCl. Samples were incubated for 1 h at RT in the elution buffer, and beads were removed using a magnetic rack. These samples were used for western blot analyses.

Two additional independent replicates of proliferating C2C12 cells were also immunoprecipitated with the anti-MTF1 antibody as described above, and after elution were eluted and subjected to in-solution digestion. These samples were analyzed at the proteomic facility at the University of Massachusetts Chan Medical School facility. Briefly, samples were reduced with 10 mM dithiothreitol (DTT) at 37°C for 30 min and alkylated with 25 mM iodoacetamide (IAA) in the dark for 45 min at room temperature. Proteins were then digested overnight at 37°C using sequencing-grade trypsin (1:50 enzyme-to-protein ratio). The digestion was quenched with formic acid, and peptides were desalted using C18 solid-phase extraction. The resulting peptides were analyzed by liquid chromatography–tandem mass spectrometry (LC–MS/MS) on a high-resolution mass spectrometer. Raw data were processed using Scaffold viewer, and protein identification was performed using the Uniprot database [55].

We then performed functional enrichment and protein–protein interaction (PPI) network analyses to characterize the MTF1 immunoprecipitated proteins. First, to standardize gene identifiers for downstream analysis, protein names were converted to Ensembl Gene IDs using g: Profiler [56]. Outdated or deprecated symbols were cross-referenced and updated using the UniProt database [55] to ensure consistent coverage in the functional enrichment analysis. Gene Ontology Molecular Function (GO: MF) enrichment analysis was performed using g: Profiler with default statistical thresholds [56]. Enriched GO terms were compared across conditions (−Cu, +Cu, and shared) to determine interactor sets of Cu-dependent *vs*. core molecular functions. PPI networks were generated using STRING-db (v11.5) [57]. Network statistics, including node and edge counts, average node degree, clustering coefficient, expected edge number, and PPI enrichment *P*-values, were computed for each condition (+Cu, +Cu, shared). These metrics provided a quantitative framework to interpret enrichment results within the broader network organization.

### Metal determinations by flame atomic absorption spectroscopy

Three independent biological replicates of control and myoblasts KD for the SWI/SNF subunits were cultured under proliferating conditions and supplemented with 100 µM CuSO_4_ and 50 µM ZnSO_4_ as described above. Cells were washed with ice-cold PBS three times, scraped, and transferred to a 1.5 ml microcentrifuge tube. Samples were sonicated using a Bioruptor at medium intensity for 5 min with 30 s on-off cycles and total protein was quantified using the Bradford method [35]. Samples were acid digested in concentrated HNO_3_ for ultra-trace analysis and diluted in purified water with a resistivity of 18 MΩ. Reagents and glassware were of analytical grade and cleaned with 3% HCl for 24 h to prevent contamination.

Comparative analysis of metal concentrations was carried out by triplicate measurements of Cu or Zn in each sample, using a flame atomic absorbance spectrophotometer (AAS; Agilent 55 AA Atomic Absorption Spectrometer) with Cu or Zn hollow cathode lamps as radiation source. Cu and Zn standard solutions (1000 mg/l; Sigma-Aldrich) were diluted as necessary to obtain working standards and determine the limits of detection and dynamic range of the method. Cu and Zn content on each sample was normalized to the initial cell mass as previously described [30, 32, 58–62].

### Visualization of labile Cu and Zn in C2C12 myoblasts using fluorescent sensors

To visualize labile Cu-pools in proliferating myoblasts we used the membrane-permeable fluorescent dyes CS1 for Cu^+^ and CD649.2 to detect Cu^2+^ following standard procedures [34, 63–66]. Control and the SWI/SNF KD myoblasts were seeded in CellView Plates and treated with or without 100 µM CuSO_4_ as described. Before Cu probe incubation, cells were washed with PBS containing GlutaMax and CaCl_2_, then incubated with the probes (5 µM) in the dark for 10 min. Imaging was done using a Leica SP8 confocal microscope and analyzed with the Leica Application Suite X. The CS1 probe visualized Cu^+^ under 543 nm excitation and 566 nm emission and the CD649.2 probe for Cu^2+^ was imaged at 633 nm excitation, and fluorescence emission was collected in the range of 650–750 nm [63–65].

To detect labile Zn^2+^ in proliferating C2C12 myoblasts, the cells were loaded with 2 μM Fluo-Zin3 AM cell permeant dye (Invitrogen, cat. No: F24195) in the corresponding culture media for 40 min at 37°C, as previously described [62]. Cells were then washed with fresh media and allowed to rest for another 20 min at 37°C. Culture media was removed; the cells were washed with fresh PBS and immediately visualized using a Leica SP8 microscope. Images were analyzed with the Leica Application Suite X. Fluo-Zin3, AM has an excitation and emission maximum of 494/516 nm.

### Statistical Analysis

All statistical analysis was performed in Kaleidagraph (Version 4.1) or Microsoft Excel. Statistical significance was determined using a Student’s *t*-test. Any experiments where *P* < .05 were considered statistically significant.

### Models and diagrams

Biological schematics were created using BioRender.com.

## Results

### *Baf180*-deficient myoblasts are sensitive to metal stress, whereas Cu and Zn supplementation rescues proliferation defects in *Baf250a* and *Brd9*-deficient cells

We previously showed that KD of *Baf250a* significantly impairs myoblast proliferation, whereas *Brd9* KD results in a more modest reduction, and *Baf180* KD has no detectable effect on proliferation under basal growth conditions [12]. Despite the lack of overt proliferative phenotype, RNA-seq analyses revealed substantial changes in the expression of genes involved in cellular homeostasis in *Baf180* KD myoblasts [12]. These observations prompted us to examine whether SWI/SNF subunits differentially contribute to myoblast responses under conditions of metal stress.

**Figure 1:**
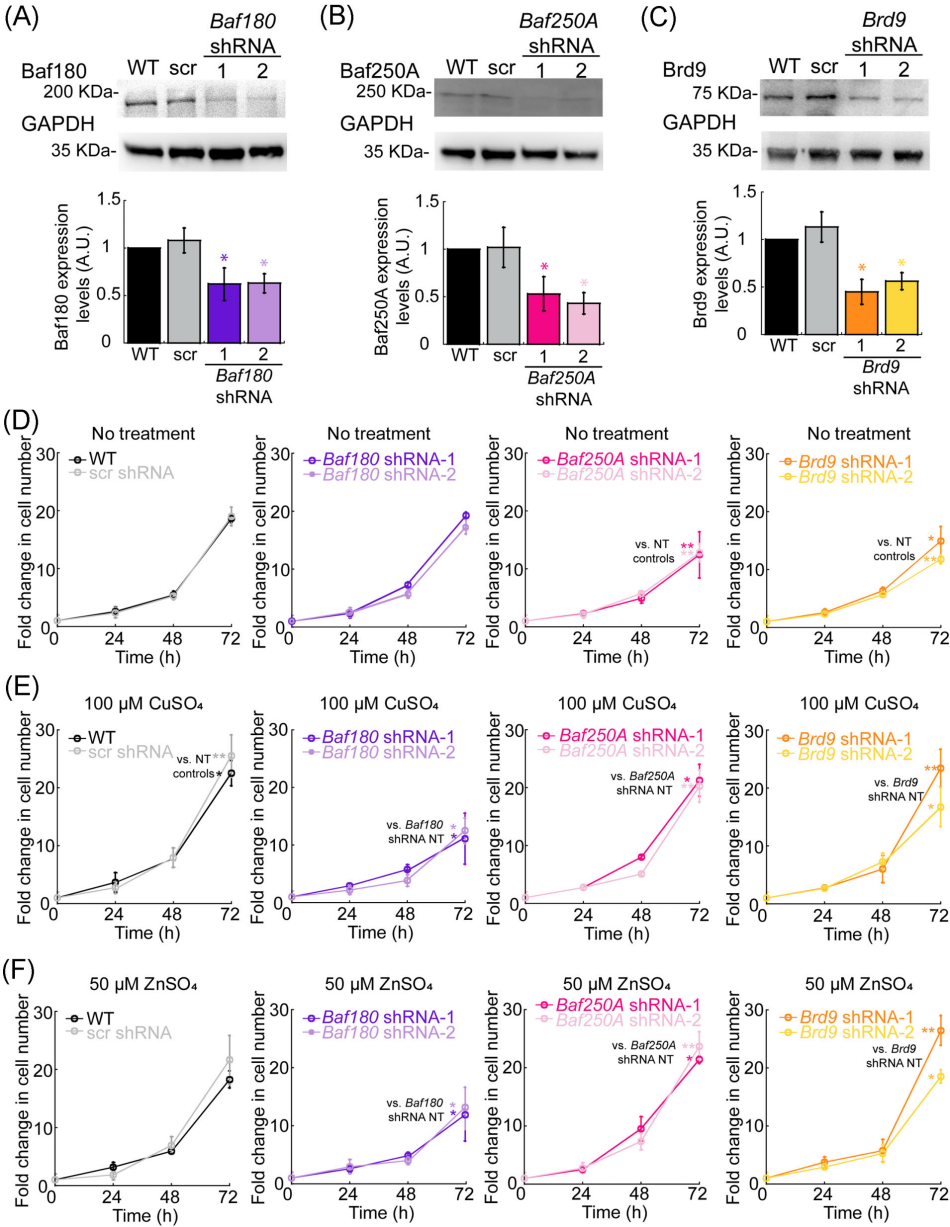
Expression and proliferation phenotype in response to metal supplementation of C2C12 myoblasts KD for *Baf180, Baf250a*, and *Brd9*. (A–C) Representative western blots (top) and quantification (bottom) of Baf180 (A), Baf250a (B), and Brd9 (C) protein levels in proliferating myoblasts, normalized to GAPDH as a loading control. Data represents the mean ± SE of three independent biological replicates. *P < .05 compared to scr control. (D–F) Proliferation analysis of WT, scr, and KD myoblasts over 72 h using cell counting assays for cells cultured under basal conditions (D) or supplemented with 100 µM CuSO_4_ (E) or 50 µM ZnSO_4_ (F). *Baf180* KD myoblasts exhibit no significant difference under basal conditions, non-treated (NT), but this cell line is sensitive to both metals. *Baf250a* KD and Brd9 KD exhibit reduced proliferation, which is rescued by metal supplementation. Data represent the mean ± SE of three independent experiments. *P < .05; **P < .01 compared to the same strain cultured in the absence of metals (NT).

To this end, we assessed the proliferative capacity of C2C12 myoblasts stably expressing shRNA targeting *Baf180, Baf250a*, or *Brd9* following exposure to Cu or Zn. Efficient protein depletion was confirmed by western blot analysis (Fig. 1A–C). Cells were cultured in the presence of increasing concentrations of CuSO_4_ and ZnSO_4_, and proliferation was evaluated by cell counting and Pax7 immunostaining, a marker of proliferating myoblasts (Fig. 1D and E; Supp. Figs 1–4). Wild-type (WT) and scrambled control (scr) myoblasts tolerated CuSO_4_ concentrations up to 100 µM and exhibited enhanced proliferation, consistent with previous reports (Fig. 1E; Supp. Figs 1 and 2; [30]). In contrast, *Baf180* KD myoblasts displayed a significant reduction in cell number at CuSO_4_ of 50 µM and above (Fig. 1E, Supp. Figs 1, 2). Notably, supplementation with 50-100 µM CuSO_4_ rescued the proliferation defects observed in *Baf250a* and *Brd9* KD myoblasts, whereas concentrations above 200 µM were toxic to all cell lines tested (Fig. 1E, Supp. Figs 1 and 2).

A similar pattern was observed upon Zn treatment. WT and scr myoblasts maintained normal proliferation at 50 µM ZnSO_4_, while *Baf180* KD myoblasts exhibited reduced proliferation at this concentration (Fig. 1F; Supp. Figs 3 and 4). In contrast, ZnSO_4_ supplementation restored the proliferation defect of *Baf250a* and *Brd9* KD myoblasts to near WT levels (Fig. 1F; Supp. Figs 3 and 4).

Based on these results, we selected 100 µM CuSO_4_ and 50 µM ZnSO_4_ for subsequent experiments, as these conditions altered proliferative outcomes without inducing cell death. Together, these data demonstrate that *Baf180* deficiency uniquely sensitizes myoblasts to metal stress, whereas Cu and Zn supplementation can compensate for proliferation defects associated with loss of *Baf250a* or *Brd9*.

### SWI/SNF subunit depletion differentially disrupts metal homeostasis in proliferating myoblasts

We have previously shown that myoblast proliferation and differentiation are accompanied by changes in intracellular metal distribution and metalloprotein expression [30, 32, 34, 62]. During myogenesis, Cu levels increase in the nucleus and cytosol, while Zn undergoes regulated efflux followed by gradual recovery as myotubes mature. Given the sensitivity of cellular processes to metal imbalance [67–70], we investigated whether depletion of SWI/SNF subunits alters metal homeostasis in proliferating myoblasts.

**Figure 2:**
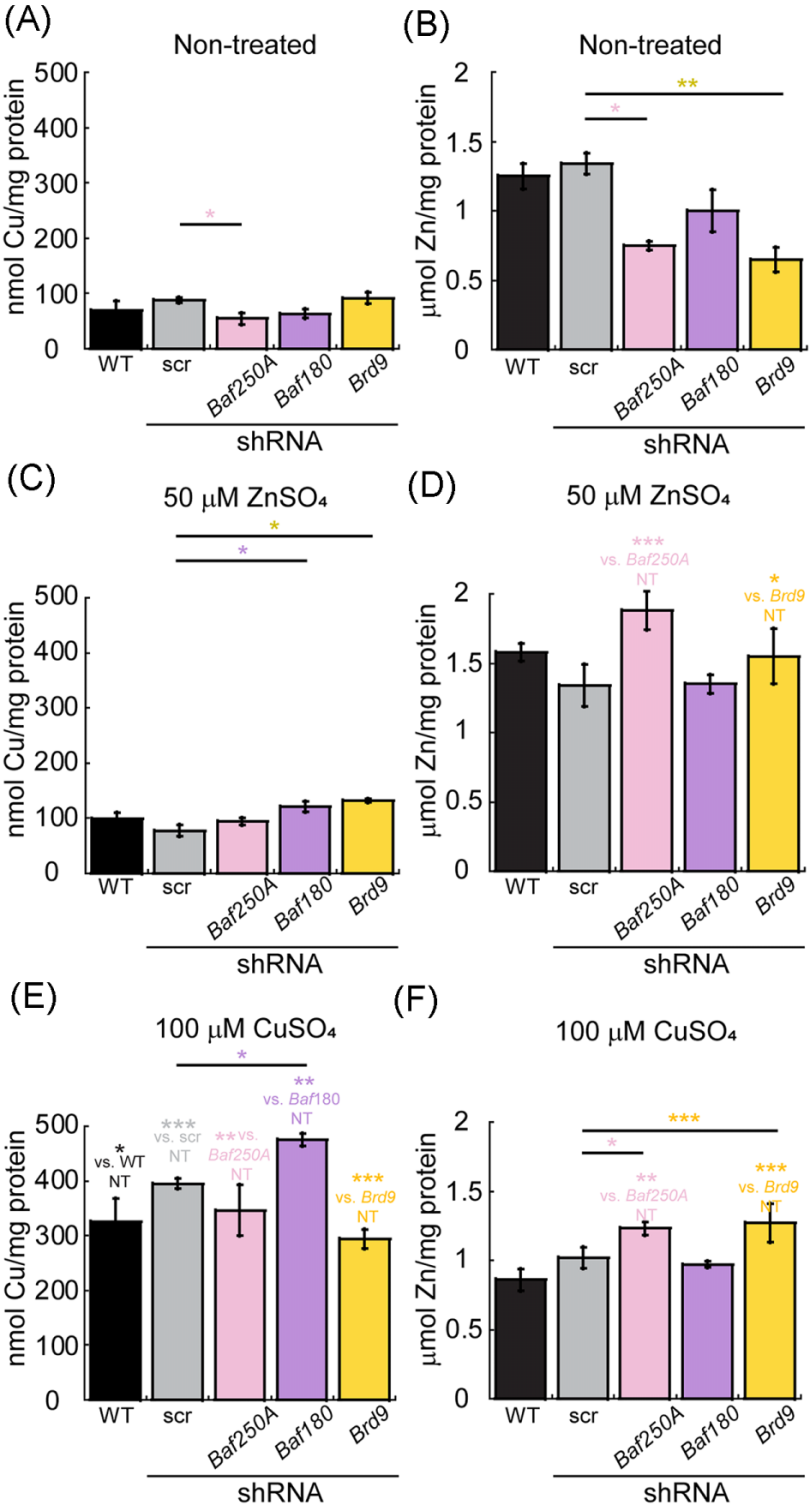
Knockdown of SWI/SNF subunits differentially disrupts Cu and Zn homeostasis in C2C12 myoblasts. Flame atomic absorption spectroscopy (AAS) analysis quantifying total intracellular Cu and Zn levels in C2C12 myoblasts under different conditions. (A) Quantification of total Cu and (B) total Zn for proliferating myoblasts cultured in basal medium without supplementation. (C) Quantification of total Cu and (D) total Zn for proliferating myoblasts cultured in the presence of 50 μM ZnSO_4_. (E) Quantification of total Cu and (F) total Zn for proliferating myoblasts cultured with 100 μM CuSO_4_. *Baf180* KD myoblasts exhibit significantly higher intracellular Cu accumulation upon CuSO_4_ supplementation, suggesting impaired Cu homeostasis. *Baf250a* KD have lower Cu and Zn content while *Brd9* KD myoblasts showed reduced levels of Zn. These defects are partially restored upon Cu or Zn supplementation. Data represents the mean ± SE of three independent experiments. Significance was obtained by comparing to scr control cells *vs*. each KD. The significant data is indicated as *P < .05; **P < .01; ***P < .001, the remaining comparisons were determined as non-significant, and were not marked in the figure for simplicity.

Total intracellular Cu and Zn levels were quantified using atomic absorption spectroscopy (AAS). Under basal conditions, *Baf250a* KD myoblasts exhibited significant reductions in both Cu and Zn relative to WT and scr controls (Fig. 2A and B). *Brd9* KD myoblasts showed a pronounced Zn deficiency, but no significant change in total Cu levels, whereas *Baf180* KD myoblasts displayed metal levels comparable to controls (Fig. 2A and B).

Upon metal supplementation, distinct patterns emerged. Treatment with CuSO_4_ or ZnSO_4_ restored Cu and Zn levels in *Baf250a* KD myoblasts to those observed in control cells and corrected Zn deficiency in both *Baf250a* and *Brd9* KD myoblasts (Fig. 2C and D). In contrast, CuSO_4_ supplementation led to a substantial accumulation of Cu in *Baf180* KD myoblasts (Fig. 2E and F), consistent with their heightened sensitivity to metal stress.

**Figure 3:**
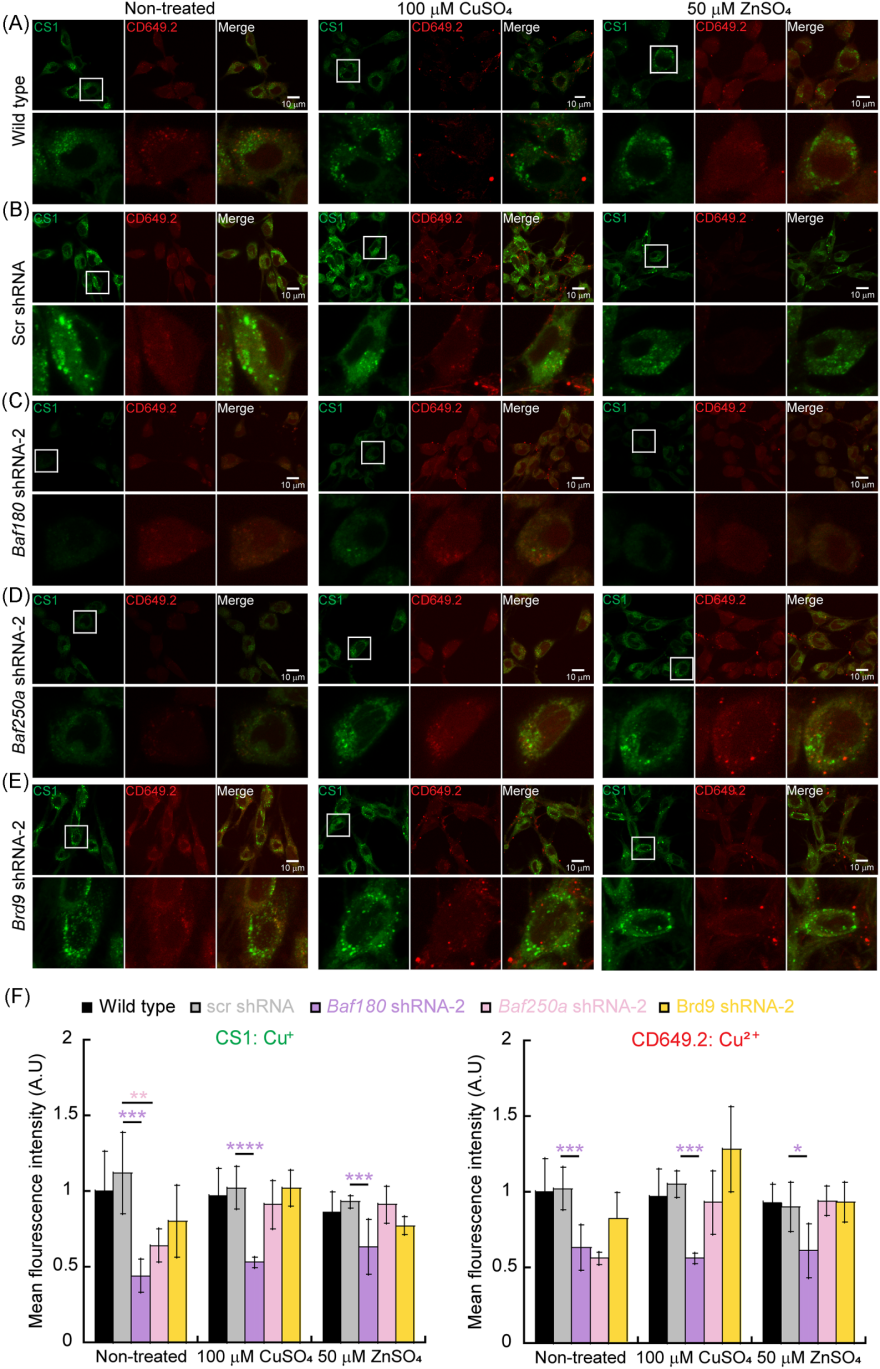
Distribution of labile Cu in control and SWI/SNF KD proliferating C2C12 myoblasts. Confocal microscopy live-cell analysis of labile Cu levels in wild-type (A), Scr control (B), and KD myoblasts for *Baf180* (C), *Baf250a* (D), and *Brd9* (E) after 48 h culture in basal untreated media (NT) or supplemented with 100 μM CuSO_4_ or 50 μM ZnSO_4._ Labile Cu⁺ was detected using CS1 probe, and Cu^2+^ was visualized using CD649.2. (F) Quantification of fluorescence from live-cell imaging: Cu^+^ with the CS1 probe (left panel) and Cu^2+^ with the CD649.2 probe (right panel) in proliferating C2C12 myoblasts. *N* = 3, *P < .05; ** *P* < .01, ***P < .001; ^****^P < .0001 when comparing each condition to the scr control. The remaining comparisons were determined as non-significant, and were not marked in the figure for simplicity. *Baf180* KD myoblasts exhibited decreased pools of labile Cu^+^ under all conditions tested, suggesting impaired copper mobilization. Scale bar: 10 μm.

Despite this accumulation, copper supplementation failed to restore proliferation in *Baf180* KD cells, suggesting that excess copper may not be effectively mobilized for cellular functions. To examine this possibility, we performed live-cell imaging using metal-specific fluorescent probes to detect labile Cu^+^ (CS1), Cu^2+^ (CD649.2), and Zn^2+^ (FluoZin3) (Fig. 3; Supp. Fig. 5). In untreated control and *Brd9* KD myoblasts, monovalent Cu was detected in discrete subcellular compartments, consistent with bioavailable Cu pools (Fig. 3A, B and E). In contrast, *Baf180* and *Baf250a* KD myoblasts exhibited reduced labile Cu⁺ or Cu²⁺ signals under basal conditions (Fig. 3C and D). CuSO_4_ restored labile Cu⁺ in *Baf250a* KD cells but failed to do so in *Baf180* KD myoblasts (Fig. 3C and D), indicating defective copper mobilization despite increased total copper levels.

To determine how disruption of distinct SWI/SNF subcomplex components alters metal-responsive fluorescence, we quantified total red fluorescence intensity from FluoZin3 images across the five genotypes. Confocal imaging (Supp. Fig. 5A) and quantitative analysis (Supp. Fig. 5B) of labile Zn revealed vesicular cytosolic pools in proliferating control myoblasts, as previously reported [62]. Control WT and scr myoblasts showed a small, non-significant decrease in labile Zn upon Cu treatment, which was not detected in *Baf180* KD myoblasts. On the other hand, the *Baf250a* and *Brd9* KD cell lines exhibited reduced labile Zn under basal conditions, which were restored to WT levels by CuSO_4_ and ZnSO_4_ supplementation. Collectively, these results suggest that disruption of distinct SWI/SNF subcomplexes differentially modulates cellular responses to Cu and Zn exposure, and supports a model of subcomplex-specific role for chromatin remodeling machinery in regulating metal-responsive cellular processes in proliferating C2C12 myoblasts.

### Baf180 exhibits colocalization and physical interaction with MTF1 in myoblasts

Our results indicate that Baf180 is dispensable for basal myoblast proliferation but is required for appropriate cellular responses to metal stress, including maintenance of metal-responsive gene expression and proliferative capacity. RNA-seq analyses revealed downregulation of metal homeostasis genes in *Baf180* KD myoblasts [12], consistent with their heightened sensitivity to Cu and Zn supplementation (Fig. 1; Supp. Figs 1–4). Given that MTF1 is a master regulator of cellular metal responses, we investigated whether MTF1 functionally associates with SWI/SNF complexes in proliferating C2C12 myoblasts.

**Figure 4:**
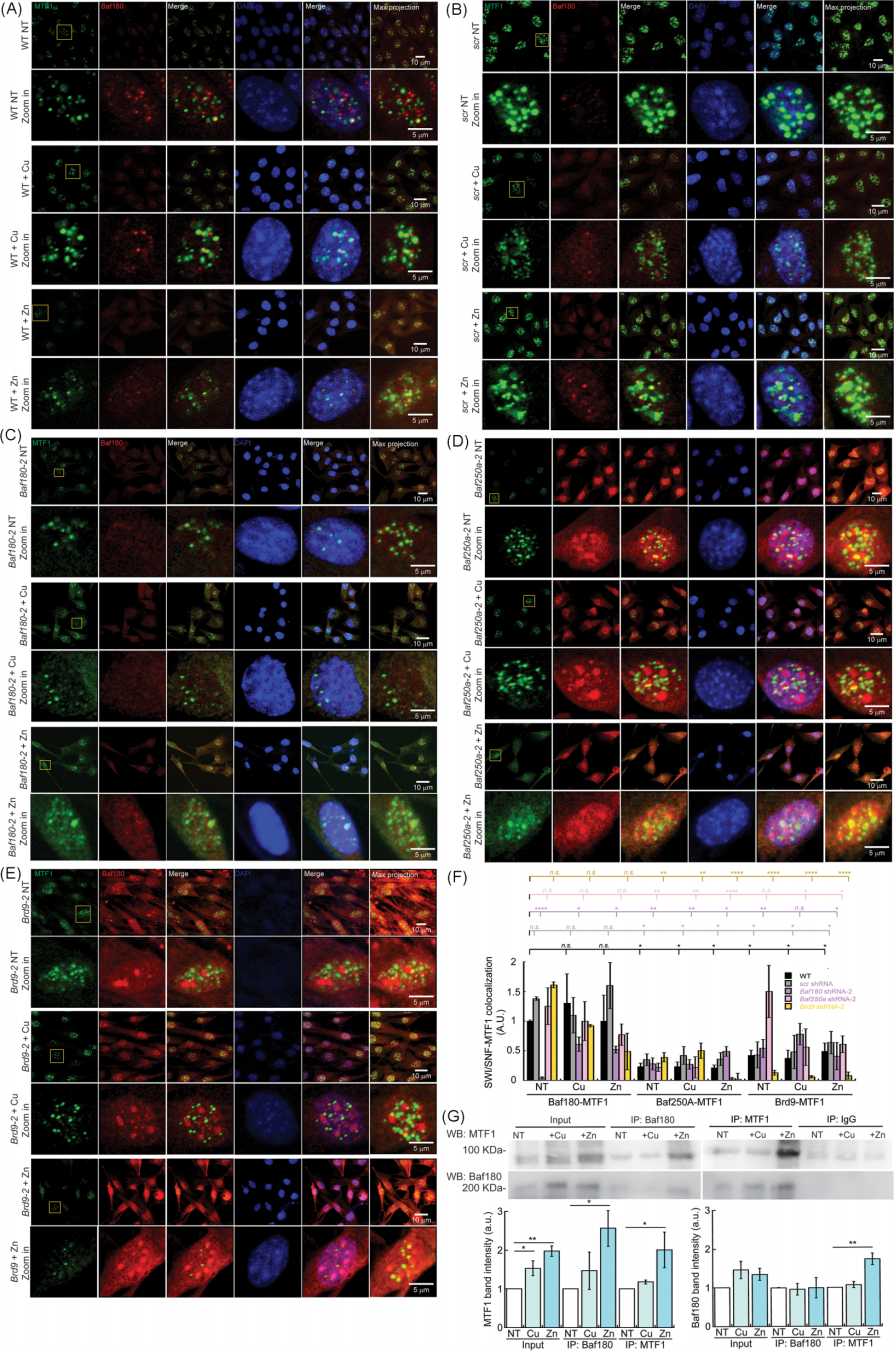
BAF180 and MTF1 colocalize and interact in C2C12 control and SWI/SNF KD myoblasts upon metal treatment. Representative confocal microscopy images showing the expression and nuclear localization of Baf180 and MTF1 in wild-type (A), scr (B), *Baf180* KD (C), *Baf250a* KD (D), and *Brd9* KD (E) C2C12 myoblasts. Nuclei are counterstained with DAPI (blue). (F) Quantification shows that MTF1 exhibits significantly higher colocalization with BAF180 in control and the different SWI/SNF KD myoblasts from panel A–E and from supplemental Figs 6 and 7. Colocalization is disrupted in *Baf180* KD cells, while metal treatment modulates MTF1-BAF180 interactions. Scale bars: 10 μm (panoramic views), 5 μm (zoomed-in images). Data represent mean ± SE of three independent biological replicates. *P < .05, **P < .01, ^****^P < .0001. * Indicates significance relative to untreated WT cells. (G) Representative immunoprecipitation (IP) and quantification of MTF1 or BAF180 in proliferating wild type myoblasts (*N* = 3). Input, a Baf180 IP obtained from *Baf180* KD cells and an IgG IP were included as controls. MTF1 exhibits significantly higher colocalization with BAF180 compared to BAF250A or BRD9 in WT proliferating myoblasts.

To assess potential interactions between MTF1 and distinct SWI/SNF subunits, we first examined protein distribution and colocalization in WT myoblasts under basal conditions and following Cu or Zn supplementation. Confocal microscopy revealed that Baf180 exhibited significantly higher colocalization with MTF1 compared to Baf250A or Brd9 (Fig. 4A, B and F; Supp. Figs 6A and B and 7A and B). This preferential colocalization was evident under untreated conditions and persisted following metal supplementation, suggesting a stable association between Baf180 and MTF1 that is not disturbed by acute metal exposure.

We next examined SWI/SNF subunit expression and MTF1 colocalization n *Baf180, Baf250a*, and *Brd9* KD myoblasts. As expected, Baf180 protein levels were significantly reduced in *Baf180* KD cells (Fig. 4C), accompanied by a significant decrease in Baf180-MTF1 colocalization across all treatment conditions (Fig. 4F). In contrast, *Baf250a* and *Brd9* KD myoblasts displayed increased Baf180 signal, frequently appearing as discrete nuclear speckles (Fig. 4D and E). Correspondingly, these cells exhibited enhanced Baf180-MTF1 colocalization relative to controls (Fig. 4F). The functional significance of these nuclear speckles remains unclear and warrants further investigation. Notably, MTF1 expression is not altered across KD conditions, indicating that changes in colocalization primarily reflect altered Baf180 abundance or distribution rather than changes in MTF1 levels (Supp. Fig. 8).

To confirm whether Baf180 and MTF1 physically associate, we performed reciprocal immunoprecipitation assays in WT myoblasts cultured in the presence or absence of Cu and Zn. Immunoprecipitation of either MTF1 or Baf180 followed by western blot analysis confirmed a specific interaction between these two proteins (Fig. 4G, top panel). Control experiments using IgG lysates verified the specificity of the interaction. Quantification of three independent immunoprecipitation experiments suggests a stronger interaction in the presence of Zn (Fig. 4G, lower panel).

To further characterize the MTF1 interactome, we performed unbiased IP-mass spectrometry (IP-MS) analyses in myoblasts cultured under Cu-deficient or Cu-supplemented conditions (Supp. Table 2 and  Supp. Fig. 9). Consistent with our microscopy and co-IP data, Baf180, Baf250a, and Brg1 were identified among MTF1-associated proteins, supporting a functional relationship between MTF1 and SWI/SNF chromatin-remodeling complexes. In contrast, Brd9 was not consistently detected, suggesting preferential engagement of canonical and PBAF complexes.

Gene ontology (GO) and protein–protein interaction (PPI) network analyses revealed that MTF1-associated proteins span diverse functional categories, including catalytic activity, nucleotide and ion-binding, oxidoreductase activity, and cytoskeletal organization (Supp. Fig. 9). Under copper-deficient conditions, the MTF1 interactome was enriched for proteins in core metabolic pathways, including amino acid metabolism, nucleotide biosynthesis, glycolysis/gluconeogenesis and the TCA cycle (Supp. Fig. 9A). Network analysis revealed a moderately connected interactome (105 nodes, 230 edges; clustering coefficient of 0.443; *P* < 1.0^−16^), consistent with engagement in metabolic resilience pathways (Supp. Fig. 9B).

In contrast, Cu supplementation substantially expanded the MTF1 interactome (124 nodes, 615 edges; clustering coefficient 0.528; *P* < 6.86^−6^), with enrichment for pathways related to nucleic acid metabolism, heterocyclic compound metabolism, and biosynthetic processes (Supp. Fig. 9B). These changes are consistent with a shift toward increased transcriptional and metabolic complexity under Cu-replete conditions.

A large core set of interacting proteins was shared across Cu-deficient and Cu-sufficient conditions forming a highly interconnected network (931 nodes, 29 122 edges; clustering coefficient 0.467; *P* < 1.0^−16^; Supp. Fig. 9C). This shared core likely represents essential cellular machinery that maintains metabolic and structural homeostasis independent of Cu availability. Notably, Baf180 (PBRM1) and Baf250a (ARID1A), but not Brd9, were present within this shared interactome.

Together, these data demonstrate that Baf180 preferentially colocalizes and physically associates with MTF1 in myoblasts and that Cu availability modulates the composition and functional bias of the MTF1 interactome. These findings support a close functional relationship between MTF1 and PBAF-associated nucleosome remodeling machinery during metal-responsive cellular states.

### Baf180 and Baf250a exhibit stronger associations with MTF1 than Brd9

RNA-seq analyses indicated that Baf250a and Brd9 are not major regulators of metal homeostasis-associated gene expression in proliferating myoblasts [12]. To directly compare their association with MTF1 relative to Baf180, we examined the localization and colocalization of Baf250a and Brd9 with MTF1 under basal and metal-treated conditions.

Immunofluorescence and colocalization analyses revealed limited overlap between Baf250a and MTF1 across all treatment conditions (Supp. Fig. 6). Efficient depletion of Baf250a was confirmed by fluorescence analyses (Supp. Fig. 8), while MTF1 expression remained unchanged, suggesting that neither *Baf250a* KD nor metal supplementation substantially affected MTF1 protein levels. Despite the generally low degree of colocalization, a detectable association between Baf250a and MTF1 was supported by unbiased MTF1 IP-MS analyses, in which Baf250a was consistently identified as an interactor (Supp. Table 2 and Supp. Fig. 9C).

We next assessed the association between Brd9 and MTF1 by confocal microscopy (Supp. Fig. 7). Brd9 expression was unchanged in WT and scr control cells, with efficient KD confirmed by western blot analysis (Supp. Fig. 8). In *Baf180* KD myoblasts treated with Cu, BRD9 protein levels modestly increased, suggesting a potential compensatory response to metal stress. However, Brd9-MTF1 colocalization remained low overall. Apparent increases in colocalization were observed in *Baf250a* KD cells were attributed to elevated background signal characteristic of this cell line and were not consistently reproducible across replicates (Supp. Fig. 7).

Together, these analyses indicate that while both Baf250A and Brd9 can associate with MTF1, their interactions are weaker and less consistent than that observed for Baf180. These findings further distinguish Baf180 as the SWI/SNF subunit most closely associated with MTF1 in proliferating myoblasts and reinforce a specific link between PBAF and metal-responsive transcriptional regulation.

### Metal supplementation induces distinct transcriptional responses in proliferating SWI/SNF-deficient myoblasts

To define how metal exposure alters transcriptional programs in proliferating myoblasts, we performed RNA-seq on scr control and *Baf180, Baf250a*, and *Brd9* KD C2C12 myoblasts treated with 100 µM CuSO_4_ or 50 µM ZnSO_4_. Principal component analysis (PCA) revealed that transcriptional signatures clustered primarily by SWI/SNF subunit depletion rather than metal treatment, with relatively modest separation between treated and untreated samples within each KD condition (Supp. Fig. 10). These data indicate that loss of SWI/SNF subunits establishes dominant transcriptional states, within which metal exposure elicits more targeted gene expression changes.

Consistent with our prior work, untreated KD myoblasts exhibited substantial transcriptional dysregulation, with *Baf250a* KD resulting in the greatest number of differentially expressed genes (DEGs; 9362), followed by *Baf180* KD (5968 DEGs), and *Brd9* KD (5345 DEGs) (Supp. Table 3 [12]). We next focused on genes responsive to metal supplementation within each KD background (Supp. Tables 4 and 5). Among the KD conditions, *Baf180* KD myoblasts exhibited the most pronounced transcriptional response to Cu exposure with 2 806 Cu-responsive DEGs, whereas Zn treatment altered 460 genes. In contrast, *Baf250a* KD cells displayed relatively few Cu-responsive genes (44 DEGs) but showed moderate response to Zn (401 DEGs). *Brd9* KD and scr control cells were minimally affected by Cu supplementation (80 DEGs), while Zn exposure affected a handful of genes (9 DEGs). In scr control myoblasts, Cu treatment induced upregulation of genes associated with oxidative stress (*Gsta4, Nqo1*), and repressed genes involved in cell adhesion such as *Islr*. Zn treatment upregulated canonical metal-responsive genes (*Mt1* and *Slc30a1*), and repressed genes involved RNA processing and mitochondrial organization (Supp. Fig. 11A and B). GO analysis highlighted oxidative stress defense and extracellular matrix remodeling in response to Cu, while Zn treatment was associated with changes in chromatin organization, cell cycle regulation, and macromolecular metabolism.

**Figure 5:**
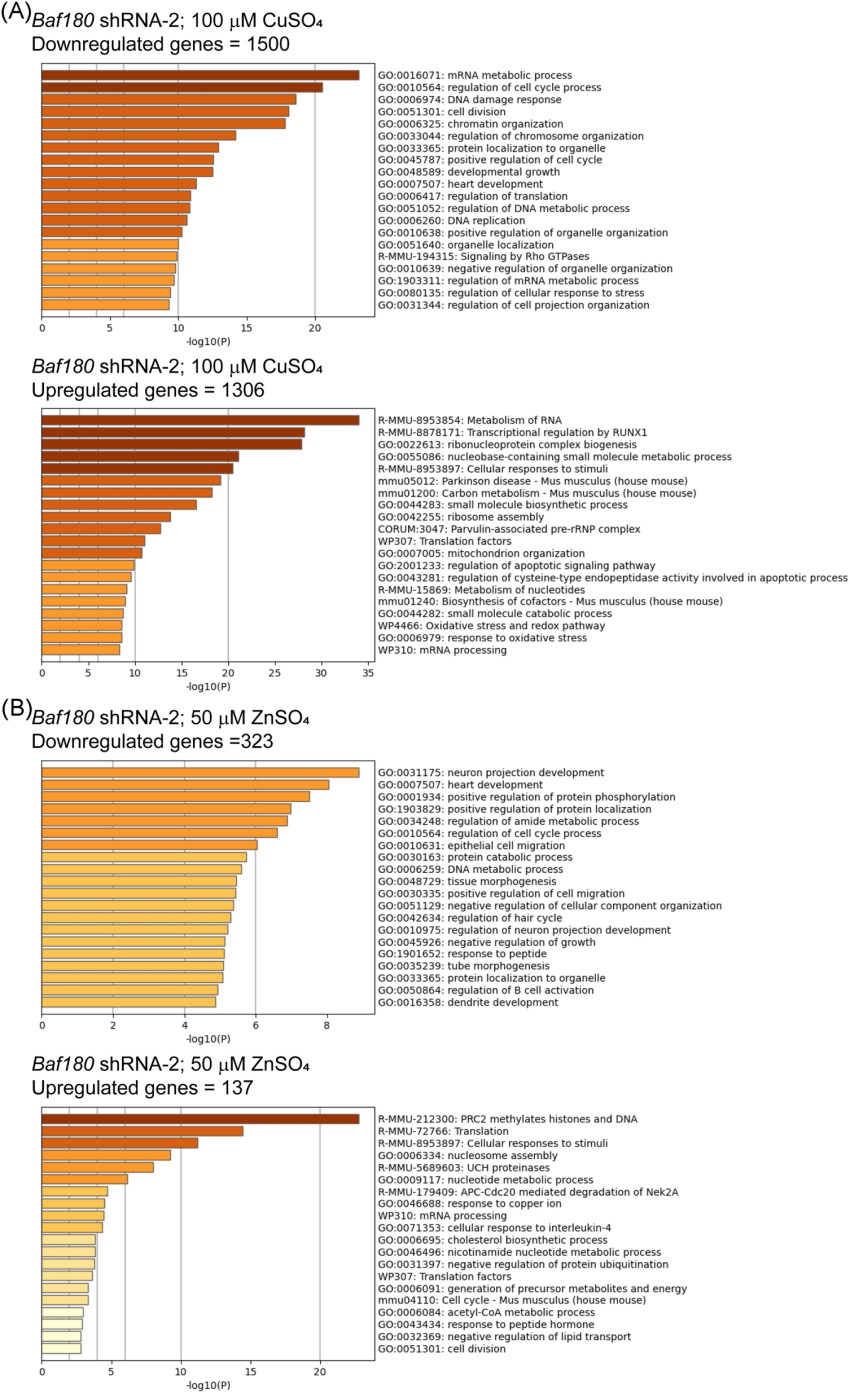
GO analysis of DEGs in *Baf180* KD myoblasts supplemented with metals. DEG identified from *Baf180* KD cells cultured in proliferation media supplemented with 100 μM CuSO_4_ (A) or 50 μM ZnSO_4_ (B) were compared to the same cell line cultured in the absence of metals (basal media). The patterns represent downregulation and upregulation of DEGs shown in Supp. Table 4.

In *Baf180* KD myoblasts metal supplementation elicited a transcriptional profile distinct from controls (Supp. Tables 4 and 5). Cu treatment downregulated genes involved in muscle regeneration and cytoskeletal organization (*Synpo2l* and *Postn*), while inducing transcription factors such as *Klf4*. GO analysis revealed repression of pathways related to mRNA metabolism, cell cycle progression, and DNA repair, alongside upregulation of mitochondrial organization and apoptotic processes (Fig. 5A). Zn treatment in *Baf180* KD cells downregulated genes associated with neuronal and cardiac development and DNA metabolism, while upregulating pathways involved in histone modification, translation, and epigenetic regulation (Fig. 5B).

In *Baf250a* KD myoblasts, Cu exposure resulted in relatively few transcriptional changes, whereas Zn supplementation significantly altered genes involved in cell cycle regulation, chromosome organization, and DNA replication (Supp. Fig 12A and **B**). Stress response genes such as *Mt1* and *Slc30a1* were induced, indicating preservation of canonical metal-responsive transcriptional programs in this context.

*Brd9* KD myoblasts exhibited minimal transcriptional response to Cu but showed a pronounced response to Zn supplementation. Zn altered expression of genes involved in cytoskeletal organization, hormone signaling, and noncoding RNA regulation, including several Rny family RNAs implicated in DNA replication and cellular stress responses (Supp. Fig 13A and **B**). As observed in *Baf250a* KD cells, Zn induced *Mt1* and *Slc30a1*, suggesting intact metal stress signaling despite ncBAF depletion.

Across KD conditions, a subset of stress-responsive genes, including *Gsta4* and *Nqo1*, was commonly induced by metal exposure, although *Nqo1* induction was attenuated in *Baf180* KD cells. Zn supplementation also induced *Wnt4* expression in *Baf250a* and *Brd9* KD myoblasts, consistent with its known role in promoting myogenic proliferation.

Notably, *Baf180* KD cells exhibited marked downregulation of *Atp7a*, a key copper exporter, following Cu treatment (Supp. Table 5). Zn exposure similarly reduced *Atp7a* expression, though to a lesser extent. In contrast, *Atp7a* expression in *Baf250a* and *Brd9* KD myoblasts was unaffected by metal exposure. Expression of the Cu importer Ctr1 (*Slc31a1*) remained stable across all conditions, indicating that altered Cu handling in *Baf180* KD cells is likely driven by impaired export rather than uptake. Together, these transcriptional data suggest that loss of Baf180 uniquely disrupts Cu responsive gene regulation, consistent with the metal accumulation and proliferation defects observed in these cells.

### MTF1 binding in proliferating myoblasts is enhanced by copper supplementation

**Figure 6:**
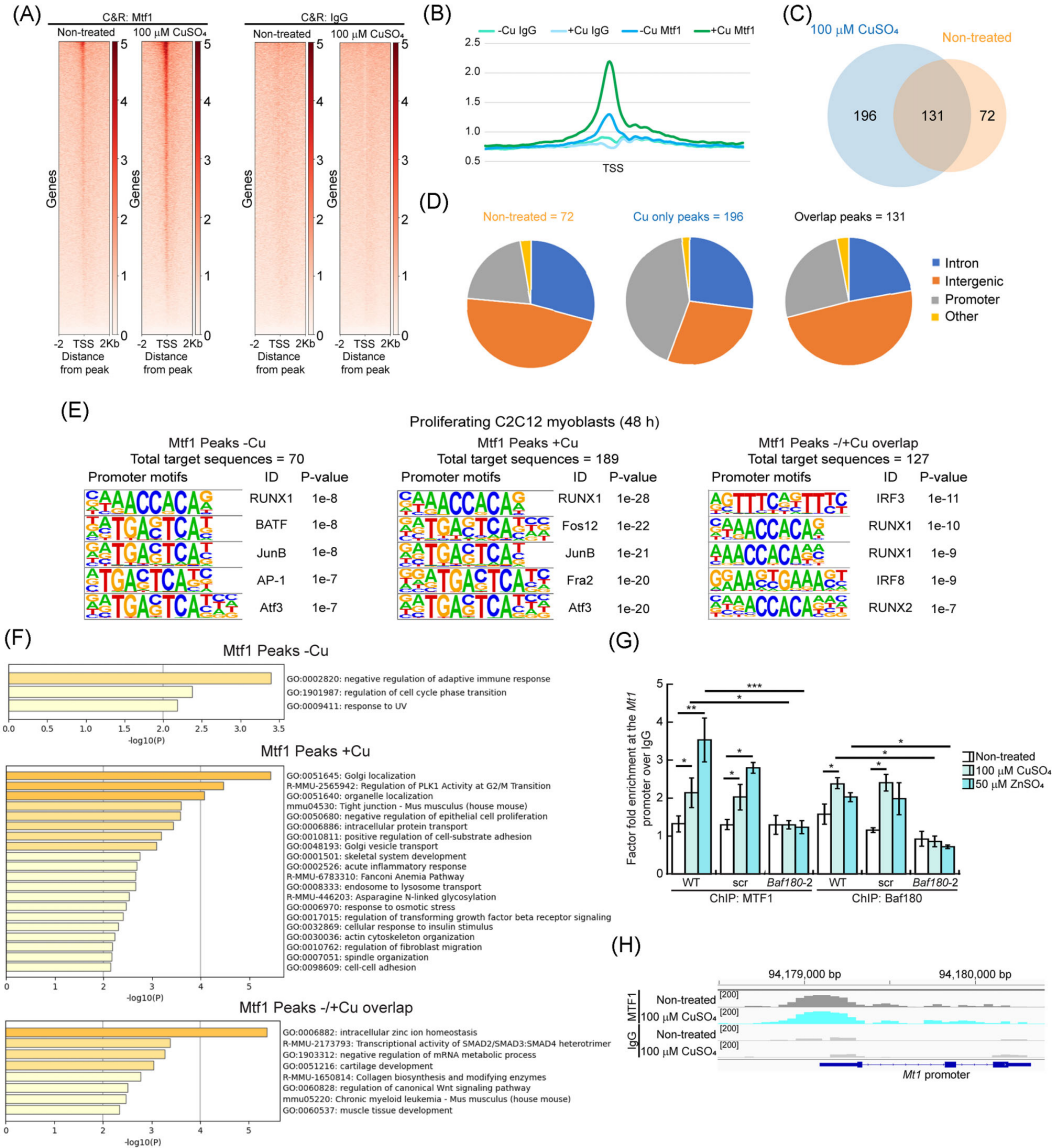
Cu supplementation enhances MTF1 chromatin binding in proliferating myoblasts. CUT&RUN analysis illustrating MTF1 binding dynamics in proliferating myoblasts under untreated conditions and following 100 μM CuSO_4_ supplementation for 48 h. (A) Combined CUT&RUN heatmaps from peak calling of three independent biological sequences obtained for each condition. Normal IgG used as control. (B) Aggregation plots of MTF1 CUT&RUN data showing occupancy of MTF1 and IgG over TSSs in the presence or absence of Cu. (C) Overlap of CUT&RUN peaks of MTF1 across the genome observed in proliferating cells in the presence or absence of Cu. See complete set of genes in Supp. Table 5. (D) Pie charts showing the binding site locations for MTF1 in myoblasts cultured in the presence or absence of Cu and combined in both conditions. (E) Main changes of MTF1 motif-binding dependent on Cu supplementation in proliferating C2C12 myoblasts. Novel consensus DNA-binding motifs identified from peak calling for CUT&RUN within MTF1 peaks in proliferating cells supplemented or not with Cu and in both conditions combined. The top five most significant motifs enriched, including the DNA logo, its corresponding transcription factor, and its *P*-value are shown. (F) GO analysis of CUT&RUN data showing the main categories of genes that MTF1 binds to in proliferating myoblasts in both the absence or presence of 100 μM CuSO_4_ and in both conditions combined. Cut-off was set at 2.0 of the -log(adjusted *P* value). Consensus DNA-binding motifs within MTF1 peaks reveal distinct sequence preferences in Cu-treated versus untreated cells. (G) ChIP-qPCR validation for MTF1 binding to the promoter region of the *Mt1* gene. (H) Genome browser tracks of the merged replicate ChIP-Seq experiments examining MTF1 binding to the *Mt1* promoter in proliferating myoblasts cultured for 48 h in the presence or absence of 100 μM CuSO_4_.

MTF1 is a transcription factor that activates gene expression in response to metal and oxidative stress [31, 71–76]. Previous studies demonstrated that MTF1 binds myogenic genes during differentiation, with Cu supplementation enhancing this binding and promoting myogenesis [32, 33]. To assess MTF1 binding in proliferating myoblasts upon Cu supplementation, we performed CUT&RUN in WT C2C12 myoblasts cultured with or without 100 μM CuSO_4_ for 48 h (Supp. Table 6). MTF1 occupancy was measured using a validated antibody, with IgG serving as a negative control. Consistent with prior observations [32], Cu supplementation significantly increased MTF1 binding at transcription start sites (TSS; Fig. 6A and B). Genome-wide peak calling analysis identified 399 promoter-proximal MTF1-bound genes, including 196 unique to Cu-treated cells, 72 unique to untreated cells, and 131 shared between conditions (Fig. 6C; Supp. Table 6). In untreated myoblasts, MTF1 binding was relatively depleted at promoters and enriched at intergenic regions, whereas Cu exposure promoted a marked redistribution toward promoter regions (Fig. 6D), consistent with enhanced transcriptional activation.

Functional annotation of MTF1-bound genes revealed enrichment for pathways involved in DNA repair, metabolism, and stress response (Supp. Table 6). Given our RNA-seq findings implicating PBAF and Baf180 in DNA damage and stress response pathways, these data raise the possibility of coordinated regulation of genome maintenance programs. Motif analysis of MTF1-bound regions identified enrichment of binding sites of immune-related transcription factors such as interferon regulatory factors (IRFs) [77] and Runx family motifs in both untreated and Cu-treated conditions (Fig. 6E). *Runx1* has been implicated in myoblast proliferation [78]. In Cu-treated cells, MTF1-bound regions were additionally enriched for AP-1 family motifs, including *Fosl2, JunB*, and *ATF3* transcriptional regulators known to cooperate with MyoD1 to promote myogenesis [79].

GO analysis further supported a role for MTF1 in regulating homeostatic and stress responsive gene programs (Fig. 6F; Supp. Table 6). In untreated myoblasts, MTF1-bound genes were associated with immune response, cell cycle regulation, and UV stress response. Cu-specific MTF1 targets were enriched for genes involved in organelle and protein localization, skeletal muscle development, and mitotic control, including pathways regulating PLK1, a key driver of cell cycle progression. Genes bound by MTF1 under both conditions were associated with transcriptional regulation, extracellular matrix organization, *Wnt* signaling, collagen synthesis, and muscle development (Fig. 6F). *Mt1* is among the classic target genes of MTF1. Consistent with previous observations from our lab from differentiating primary myoblasts [32], we detected MTF1 binding at this promoter on cells grown in the presence and absence of Cu and Zn (Fig. 6G). Importantly, this binding was reduced in myoblasts KD for *Baf180*, supporting a mechanistic interaction among MTF1 and Baf180, to support the expression of this metalloprotective gene. This data is also consistent with tracks obtained from CUT&RUN analyses (Fig. 6H). Together, these data demonstrate that Cu supplementation enhances MTF1 binding at promoters in proliferating myoblasts, similarly to our observations in differentiating cells [32], expanding its engagement with transcriptional programs linked to stress response, metabolism, and cell cycle regulation.

### Interaction of MTF1 chromatin binding with SWI/SNF-dependent transcriptional changes

To examine potential links between MTF1 chromatin binding and SWI/SNF-dependent transcriptional regulation, we integrated MTF1 CUT&RUN data from Cu-treated myoblasts with RNA-seq datasets scr control and SWI/SNF subunit KD cells under metal-treated and untreated conditions (Supp. Tables 7 and 8; Supp. Fig. 14). This analysis focused on genes that were bound by MTF1 in Cu-treated proliferating myoblasts and exhibited altered expression in response to Cu supplementation in each KD background. Across all merged datasets, the overlap between MTF1-bound genes and metal-responsive transcripts was limited, precluding robust GO analysis.

In *Baf180* KD myoblasts, Cu-induced MTF1-bound genes included transcription and signaling regulators (*Map3k3, Mafk, Jun, Ncln, Nelfcd*), components of the protein synthesis and mitochondrial machinery (*Mrpl45, Rps5, Rpl37, Mrpl18, Rps19*), and metabolic enzymes (*Got1, Eno1, Pgd, Pkm, Gsto1*). Additional Cu-induced targets included genes involved in membrane organization (*Cdc37, Plec*) and stress response (*Parp3, Dcaf15, Dusp6*). In contrast, Cu-repressed MTF1-bound genes in this KD included *Abhd17a*, involved in lipid metabolism, as well as transcription and RNA processing regulators such as *Snd1*. Several genes involved in cytoskeletal organization and signaling (*Fiz1, Ankrd2*) were also downregulated. A subset of MTF1-bound genes, including those involved in metabolism (*Ndufv3, Gstm1, Acaa1a, Sdhb*), RNA regulation (*Ppp1r14b, Khsrp, Snhg1*), cell structure (*Podnl1, Fkbp10, Lmna, Vim, Vcp*), and signaling (*Ppib, Htra2, Stub1, Eif5a*), remained Cu-independent in *Baf180* KD cells.

In *Baf250a* KD myoblasts, Cu-induced MTF1-bound genes included *Pgd* and *Txnrd1*, which contribute to NADPH production and redox balance, respectively. No MTF1-bound genes were significantly repressed by Cu in this context, while detoxification-related genes such as *Gstm1* remained Cu-independent. Similarly, in *Brd9* KD myoblasts, Cu supplementation induced MTF1-bound genes associated with redox (*Pgd, Txnrd1*) and chromatin organization (*Hmga2*). Metal homeostasis genes (*Mt1, Mt2*) and detoxification gene *Gstm1* remained largely Cu-independent, indicating preserved basal MTF1 activity despite ncBAF depletion.

Collectively, these analyses reveal that MTF1-bound genes exhibit distinct Cu-responsive expression patterns depending on SWI/SNF subunit context. While overlap between chromatin binding and transcriptional output is limited, the data suggests preferential coordination between MTF1 and the PBAF complex in shaping Cu-responsive transcriptional programs. These findings highlight the complexity of metal-regulated gene expression and underscore the importance of chromatin context in determining MTF1 transcriptional outcomes.

## Discussion

### Distinct roles of SWI/SNF subcomplexes in myoblast proliferation and metal responsiveness

SWI/SNF complexes are essential regulators of transcription programs that govern cell proliferation, differentiation, and stress responses [6, 7]. Although cBAF, PBAF, and ncBAF share a conserved ATPase core, their distinct subunit compositions suggest specialized functions that are context dependent [11]. Our previous work established that the cBAF complex is required for myoblast proliferation and differentiation through direct regulation of myogenic transcription factors such as Pax7 and Myogenin, whereas ncBAF plays a more indirect role in myogenesis, likely through effects on RNA metabolism [12, 13]. In contrast, PBAF appeared largely dispensable for core myogenic gene expression under basal conditions, although transcriptomic analyses hinted at a potential role in stress-related pathways, including metal homeostasis [12].

These results suggest that PBAF, via Baf180, supports myoblast proliferation under metal stress. Unlike BAF or ncBAF depletion, loss of Baf180 renders myoblasts uniquely sensitive to Cu and Zn perturbation, resulting in impaired proliferation. These findings highlight functional divergence among SWI/SNF subcomplexes and identify PBAF as a critical regulator of metal-responsive transcription programs rather than canonical myogenic differentiation pathways.

### PBAF uniquely supports metal-responsive gene expression and cellular resilience

Our data indicated that Baf180 is required for appropriate cellular responses to Cu and Zn in proliferating myoblasts. *Baf180*-deficient cells exhibit intracellular Cu accumulation accompanied by a reduction in labile Cu⁺ pools, suggesting defective Cu mobilization or utilization. The transcriptional profile of these cells supports impaired Cu handling, including reduced expression of *Atp7a*, a Cu exporter critical for maintaining Cu homeostasis. In addition, stress-response genes such as *Mt1* and *Gsta4*, which function in metal sequestration and oxidative stress defense, fail to be properly induced in *Baf180*-deficient cells under Cu stress.

Together, these observations suggest that PBAF supports a coordinated transcriptional response that enables myoblasts to buffer metal-induced stress and maintain proliferative capacity. In the absence of Baf180, this adaptive response is compromised, potentially leading to accumulation of non-bioavailable copper, heightened cellular stress, and impaired cell cycle progression. Although we did not directly assess DNA damage or oxidative stress in this study, the altered expression of genes involved in these pathways is consistent with increased vulnerability to metal-induced toxicity.

### Metal supplementation rescues proliferation defects of *Baf250a* and *Brd9* KD myoblasts

In contrast to *Baf180*-deficient cells, myoblasts depleted of *Baf250a* or *Brd9* exhibit proliferation defects when cultured in basal growth media [12], which can be partially rescued by Cu or Zn supplementation. These findings suggest that while cBAF and ncBAF complexes contribute to proliferation under basal conditions, they are not strictly required for metal responsive transcriptional adaptation. The mechanisms underlying metal-mediated rescue in these contexts remain unresolved. However, one possibility is that metal supplementation restores transcription of specific genes that are indirectly affected by loss of cBAF or ncBAF function, For example, *Wnt4* expression was induced by Cu in both *Baf250a* and *Brd9* knockdown cells and may contribute to restored proliferation, as WNT signaling has well-established roles in myoblast growth and differentiation [80]. Alternatively, metal supplementation may improve cellular fitness through broader metabolic or antioxidant effects, independent of nucleosome remodeling. Cu and Zn are essential cofactors for enzymes involved in redox homeostasis, mitochondrial function, and cell cycle, including superoxide dismutases (SOD1/3) and p53-associated pathways [68, 81–85] and could therefore buffer the consequences of chromatin perturbation. Another possibility is that PBAF may be required to properly distribute excess metals. These hypotheses are not mutually exclusive, and our data does not yet distinguish between transcription-specific rescue and more general metabolic or stress-buffering effects.

### MTF1 binding requires appropriate chromatin context for transcriptional output

A key finding of this study is the interaction between Baf180 and MTF1, a metal-responsive transcription factor essential for myoblast differentiation and metal homeostasis. We show that Baf180 and MTF1 colocalize and physically interact in the nucleus in the presence or absence of metal stress. These observations raise an important conceptual point: transcription factor binding alone is insufficient to ensure transcriptional output without an appropriate chromatin context. Our data suggest that PBAF may provide such a context at metal-responsive loci, enabling MTF1 to activate target gene expression effectively. In the absence of Baf180, MTF1 binding may be unstable, improperly localized, or transcriptionally unproductive, resulting in impaired induction of metal-responsive genes despite the presence of metal stress signals.

CUT&RUN analysis revealed enrichment of RUNX motifs at MTF1-bound regions, consistent with known roles for RUNX factors in myogenesis and stress responses. While these findings suggest potential cooperation between MTF1 and lineage-specific transcription factors, co-occupancy and functional interactions remain to be experimentally validated.

### A provisional model for PBAF function in metal-responsive transcription

Based on our findings, we propose a provisional model in which PBAF, through Baf180, supports metal-responsive gene expression and myoblast proliferation by facilitating effective MTF1 function under conditions of Cu or Zn stress. However, our data does not yet distinguish between several non-mutually exclusive mechanisms. PBAF may directly recruit MTF1 to chromatin, stabilize MTF1 protein levels during metal stress, or indirectly influence MTF1 activity through broader effects on transcriptional or cellular stress states. Additional work will be required to define the precise molecular relationship between PBAF and MTF1 and to determine how nucleosome remodeling intersects with metal sensing at specific genomic loci.

### Broader implications for PBAF in cellular stress responses

Our findings align with a growing body of evidence implicating PBAF in cellular stress adaptation across biological contexts. Baf180 has been shown to regulate cell cycle progression in hematopoietic stem cells [23], promote resistance to thermal stress in *C. elegans* [86], and modulate tumor suppressive pathways in human cancers, where its loss compromises stress responses and genomic stability [87]. Together with our findings, these studies support a broader role for PBAF in coordinating transcriptional responses to environmental and metabolic stressors.

In the context of skeletal muscle biology, our work positions PBAF as a key regulator of metal homeostasis and proliferative capacity in myoblasts. Given the importance of copper and zinc in muscle development and disease, dysregulation of this pathway may contribute to muscle pathologies associated with altered micronutrient availability or chromatin remodeling defects.

## Conclusion

In summary, this study identifies a previously unrecognized role for the PBAF complex in coordinating metal-responsive transcription and myoblast proliferation. We show that the PBAF subunit Baf180 uniquely sensitizes myoblasts to copper and zinc stress, leading to impaired proliferation that cannot be rescued by metal supplementation. In contrast, proliferation defects associated with cBAF or ncBAF depletion can be partially rescued by metals, highlighting the functional divergence among SWI/SNF subcomplexes.

Our data supports a model in which Baf180 interacts with MTF1 and promotes effective transcriptional responses to metal stress. Our data suggests that Baf180 supports MTF1 stability and/or may facilitate its chromatin association, although direct mechanistic evidence for recruitment remains to be established. Importantly, our findings emphasize that transcription factor binding alone is insufficient for transcriptional activation without appropriate chromatin remodeling, highlighting PBAF as a critical determinant of transcriptional competence at metal-responsive loci.

Collectively, these results establish PBAF as an important regulator of metal homeostasis and proliferative capacity in skeletal muscle progenitors and suggest that nucleosome remodeling plays a central role in integrating environmental signals with lineage-specific transcriptional programs. Future studies aimed at defining the molecular mechanisms by which PBAF modulates MTF1 activity and metal-responsive gene networks will provide deeper insight into how chromatin dynamics shape cellular adaptation to metabolic and environmental stress in development and disease.

## Supplementary Material

mfag019_Supplemental_Files

## Data Availability

Genomic data sets are deposited in the Gene Expression Omnibus (GEO) accession no. GSE253379.
