# Supplementary material for: The PBAF chromatin remodeling complex contributes to metal homeostasis through MTF1 regulation
Source: Metallomics. 2026 Jun 5;18(1):mfag019. doi: 10.1093/mtomcs/mfag019 (PMC13294679; doi:10.1093/mtomcs/mfag019)
Supplement: mfag019_Supplemental_Files [file mfag019_supplemental_files.zip › CARULLI SUPP. FIGURES resubmission (1).docx]

**The PBAF chromatin remodeling complex contributes to metal homeostasis through MTF1 regulation**

**Nick Carulli^1^**^¥^**^$^, Emma E. Johnston^1#$^, David C. Klein^2^, Odette Verdejo-Torres^1&^, Anand Parikh^1%^, Arianna McDaniels^1^, Antonio Rivera^1^, Michael Quinteros^1@^, Aidan T. Pezacki^3,4^, Christopher J. Chang^3,4,5^, Sarah J. Hainer^2^, Teresita Padilla-Benavides^1^*.**

^1^ Department of Molecular Biology and Biochemistry, Wesleyan University, CT, 06459. USA

^2^ Department of Biological Sciences. University of Pittsburgh, Pittsburgh, PA, 15260. USA

^3^ Department of Chemistry, Princeton University, Princeton, NJ, 08544. USA

^4^ Department of Chemistry, University of California, Berkeley, CA, 94720. USA

^5^ Department of Molecular and Cell Biology, University of California, Berkeley, CA 94720. USA

^¥^ Current affiliation: Department of Molecular and Cellular Biology, SUNY Downstate Health Sciences University, Brooklyn, NY, 11203 USA

^#^ Current affiliation: Tisch Multiple Sclerosis Research Center of New York, New York, NY, 10019, USA

^&^ Current affiliation: Department of Molecular, Cell and Cancer Biology, University of Massachusetts Medical School, Worcester, MA, 01605, USA

^%^ Current affiliation: Department of Computational Medicine & Bioinformatics, University of Michigan, Ann Arbor, MI, 48109, USA

^@^ Current affiliation: Department of Molecular Biology and Genetics, The Johns Hopkins University School of Medicine, Baltimore, MD, 21205, USA

^$^ These authors contributed equally to this project.

* Corresponding author.

N.C. Email: [ncarulli@wesleyan.edu](mailto:ncarulli@wesleyan.edu) ORCID: 0000-0002-7655-2966

E.E.J. Email: [eejohnston@wesleyan.edu](mailto:eejohnston@wesleyan.edu) ORCID: 0009-0004-6799-9397

D.C.K. Email: [dck28@pitt.edu](mailto:dck28@pitt.edu) ORCID: 0000-0002-0250-809X

O.V.-T. Email: [odette.verdejo@umassmed.edu](mailto:odette.verdejo@umassmed.edu) ORCID: 0009-0006-6955-5428

A.P. Email: [aparikh02@wesleyan.edu](mailto:aparikh02@wesleyan.edu) ORCID: 0009-0001-1752-5378

A.MD. Email: [amcdaniels@wesleyan.edu](mailto:amcdaniels@wesleyan.edu) ORCID: 0009-0002-0788-2387

A.R. Email: [agrivera@wesleyan.edu](mailto:agrivera@wesleyan.edu) ORCID: 0009-0001-3489-3148

M.Q. Email: [mquint10@jh.edu](mailto:mquint10@jh.edu) ORCID: 0000-0002-0779-2026

A.T.P. Email: [apezacki@scripps.edu](mailto:apezacki@scripps.edu) ORCID: 0000-0002-7321-462X

C.J.C. Email: [chrischang@princeton.edu](mailto:chrischang@princeton.edu) ORCID: 0000-0001-5732-9497

S.J.H. Email: [sarah.hainer@pitt.edu](mailto:sarah.hainer@pitt.edu); ORCID: 0000-0003-0503-1183

T.P.-B.: Email: [tpadillabena@wesleyan.edu](mailto:tpadillabena@wesleyan.edu); ORCID: 0000-0002-4624-0822

**SUPPLEMENTAL MATERIALS**

**TABLE OF CONTENTS**

**SUPPLEMENTAL REFERENCES**

Supplemental Figure 1. Cu supplementation modulates myoblast proliferation in SWI/SNF KD cells.

Supplemental Figure 2. Zn restores proliferation in *Baf250a* and *Brd9* KD myoblasts but inhibits *Baf180* KD cells.

Supplemental Figure 3. CuSO_4_ supplementation impairs *Baf180* KD myoblast proliferation but restores the proliferation defect in *Baf250A* and *Brd9* KD myoblasts.

Supplemental Figure 4. ZnSO_4_ supplementation impairs *Baf180* KD myoblast proliferation but restores the proliferation defect of *Baf250A* and *Brd9* KD myoblasts.

Supplemental Figure 5. Distribution of labile Zn in control and SWI/SNF KD proliferating C2C12 myoblasts.

Supplemental Figure 6. Expression and nuclear localization of Baf250a in proliferating C2C12 myoblasts.

Supplemental Figure 7: Expression and nuclear localization of Brd9 in proliferating C2C12 myoblasts.

Supplemental Figure 8. Quantification of SWI/SNF subunit and MTF1 protein levels in proliferating C2C12 myoblasts.

Supplemental Figure 9. Cu-dependent reorganization of the MTF1 interactome revealed by IP-mass spectrometry and PPI network analysis.

Supplemental Figure 10. Principal component analysis reveals consistent global transcriptome profiles across conditions.

Supplemental Figure 11. GO analysis of DEGs in scr control myoblasts supplemented with metals.

Supplemental Figure 12. GO analysis of DEGs in Baf250a KD myoblasts supplemented with metals.

Supplemental Figure 13. GO analysis of DEGs in Brd9 KD myoblasts supplemented with metals.

Supplemental Figure 14. MTF1 chromatin binding correlates with DEGs in SWI/SNF KD myoblasts under Cu exposure.

**SUPPLEMENTAL TABLES**

Supplemental Table 1. Plasmids used in this study

Supplemental table 2. MTF1 IP-MS data.

Supplemental table 3. RNAseq. DEG SCR vs BAF subunits KD +/- metals.

Supplemental table 4. Summary of DEG genes from metal-treated KD and scr control cells.

Supplemental table 5. RNAseq. DEG within BAF subunits KD strains +/- metals.

Supplemental Table 6. CUT&RUN. MTF1 Annotated peaks and motifs. All conditions.

Supplemental Table 7. CUT&RUN – RNAseq integration. MTF1 supplemented with Cu integrated with RNAseq comparison between similar metal treatment of KD vs SCR analyses.

Supplemental Table 8. CUT&RUN – RNAseq integration. MTF1 supplemented with Cu integrated with RNAseq comparison of Cu treatment within strains analyses.

**SUPPLEMENTAL REFERENCES**

**SUPPLEMENTAL FIGURES**

**SUPPLEMENTAL FIGURE 1**

**
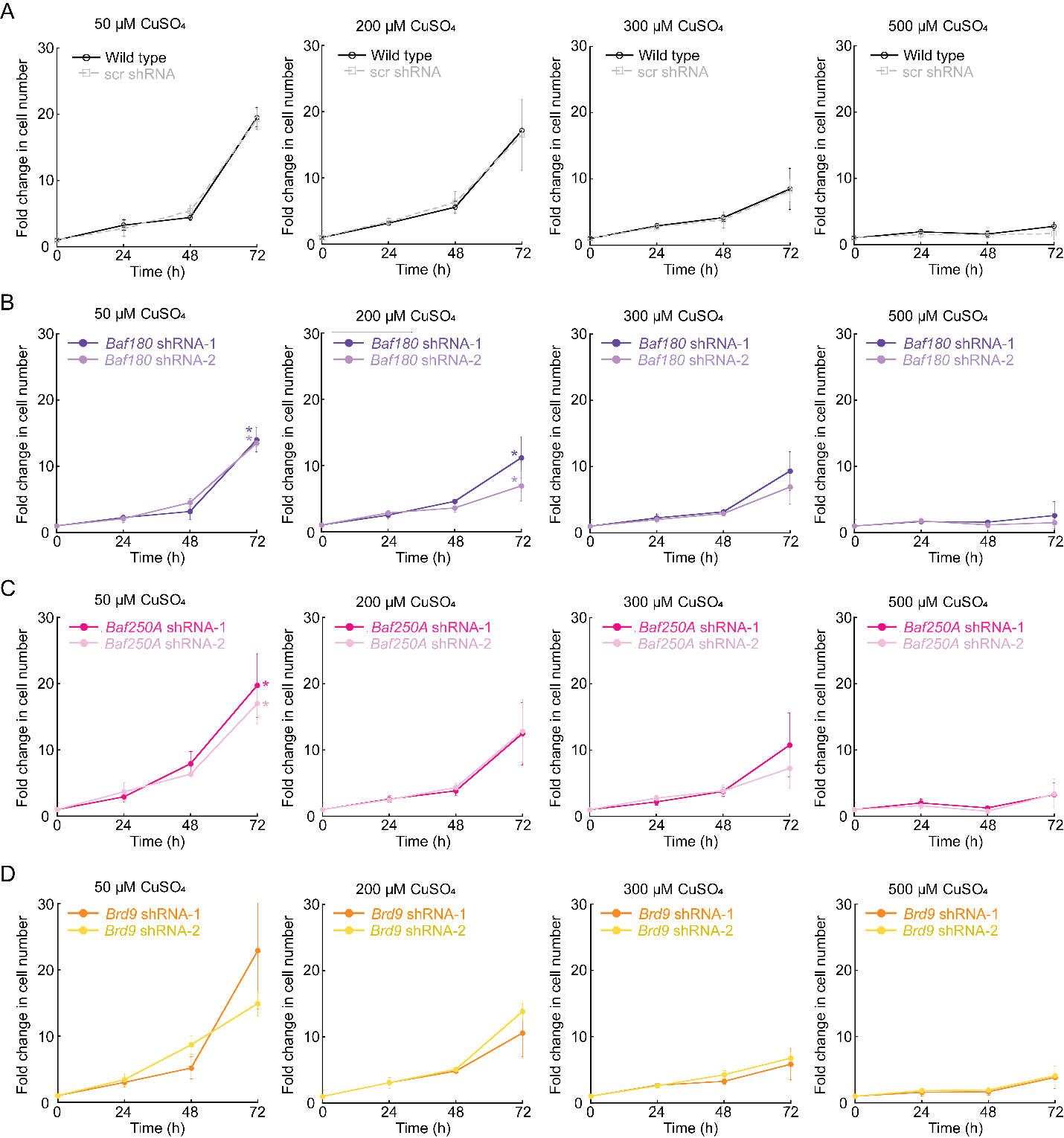
**

**Supplemental Figure 1. Cu supplementation modulates myoblast proliferation in SWI/SNF KD cells.** Cell counting assay of proliferating of C2C12 myoblasts treated with 50, 200, 300, and 500 μM CuSO_4_ over 72 h. **(A)** Wild type and scrambled shRNA (scr) C2C12 myoblasts tolerate CuSO₄ concentrations up to 200 μM. **(B)** *Baf180* KD myoblasts exhibit reduced proliferation upon CuSO_4_ treatment. **(C)** *Baf250a* KD myoblasts regain wild type-like proliferation with CuSO_4_ supplementation. **(D)** *Brd9* KD myoblasts also recover wild type-like proliferation rates with CuSO_4_ supplementation. See **Figure 1** for non-treated and 100 µM CuSO_4_ concentrations, as these were the selected conditions used in this paper. The results indicate that Cu availability differentially affects myoblast proliferation, inhibiting *Baf180*-deficient cells while restoring proliferation in *Baf250a* and *Brd9* KD myoblasts. Data represents the mean ± SE of three independent experiments. *P < 0.05 compared to the same strain cultured in the absence of metals (NT).

**SUPPLEMENTAL FIGURE 2**

**
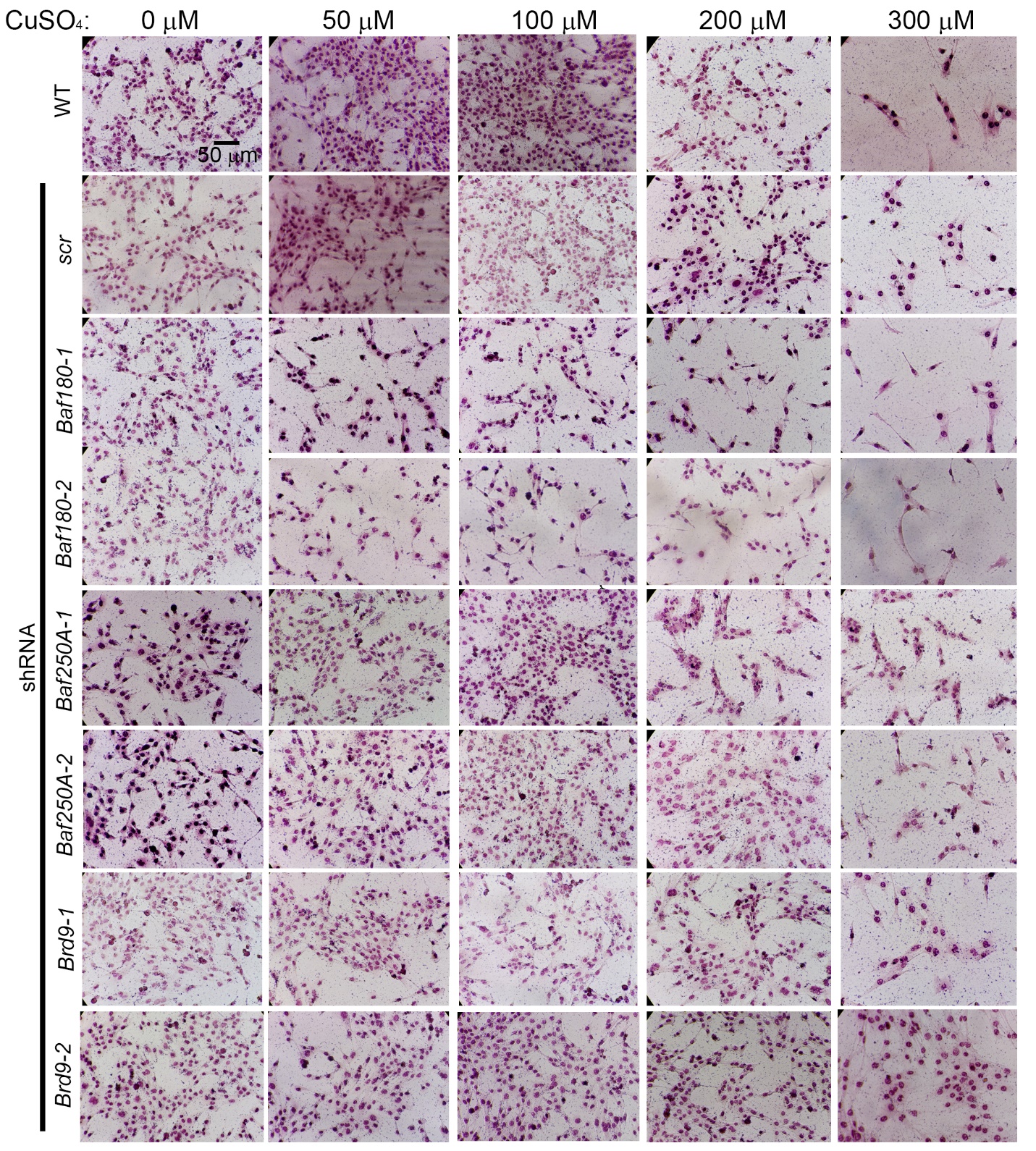
**

**Supplemental Figure 2. CuSO_4_ supplementation impairs *Baf180* KD myoblast proliferation but restores the proliferation defect in *Baf250A* and *Brd9* KD myoblasts.** Representative light micrographs of proliferating WT, scr, *Baf180* KD*, Baf250A* KD*,* or *Brd9* KD C2C12 myoblasts supplemented with 0, 50, 100, 200, and 300 μM CuSO4 over 48 h and immunostained for Pax7.

**SUPPLEMENTAL FIGURE 3**

**
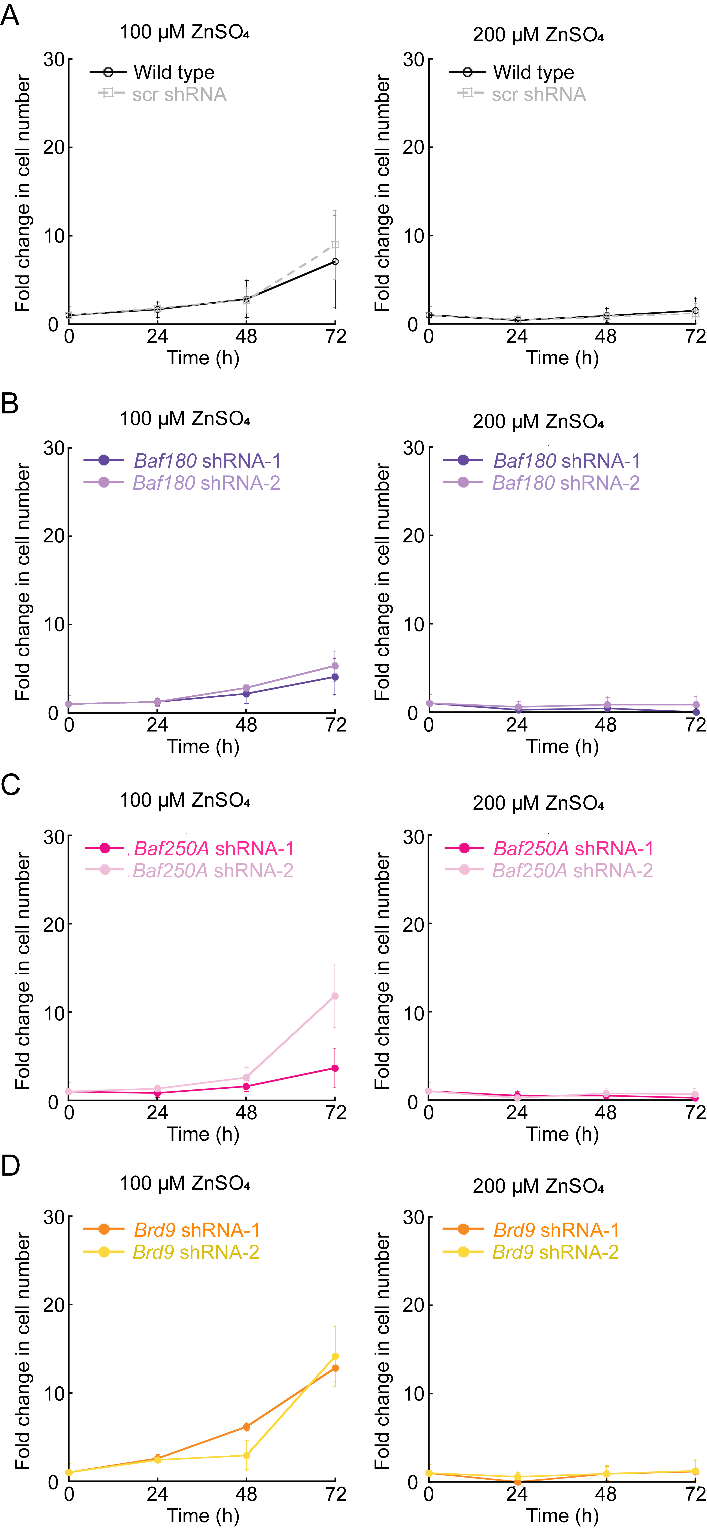
**

**Supplemental Figure 3. Zn restores proliferation in *Baf250a* and *Brd9* KD myoblasts but inhibits *Baf180* KD Cells.** Cell counting assay of proliferating of C2C12 myoblasts treated with 100 and 200 μM ZnSO_4_ over 72 h. **(A)** Wild type and scrambled shRNA (scr) C2C12 myoblasts are sensitive to ZnSO_4_ over 50 µM as previously shown (1). *Baf180* **(B)** and *Baf250a* **(C)** KD myoblasts exhibit reduced proliferation upon ZnSO_4_ treatment. **(D)** *Brd9* KD myoblasts also recover wild type-like proliferation rates with ZnSO_4_ supplementation. See **Figure 1** for non-treated and 50 µM ZnSO_4_ concentrations, as these were the selected conditions used in this paper. These data suggest that Zn supplementation restores proliferation defects in *Baf250a*- and *Brd9*-deficient myoblasts at low concentrations and negatively impacts *Baf180* KD cells, highlighting distinct roles for SWI/SNF subunits in metal homeostasis and muscle cell growth. Data represents the mean ± SE of three independent experiments.

**SUPPLEMENTAL FIGURE 4**


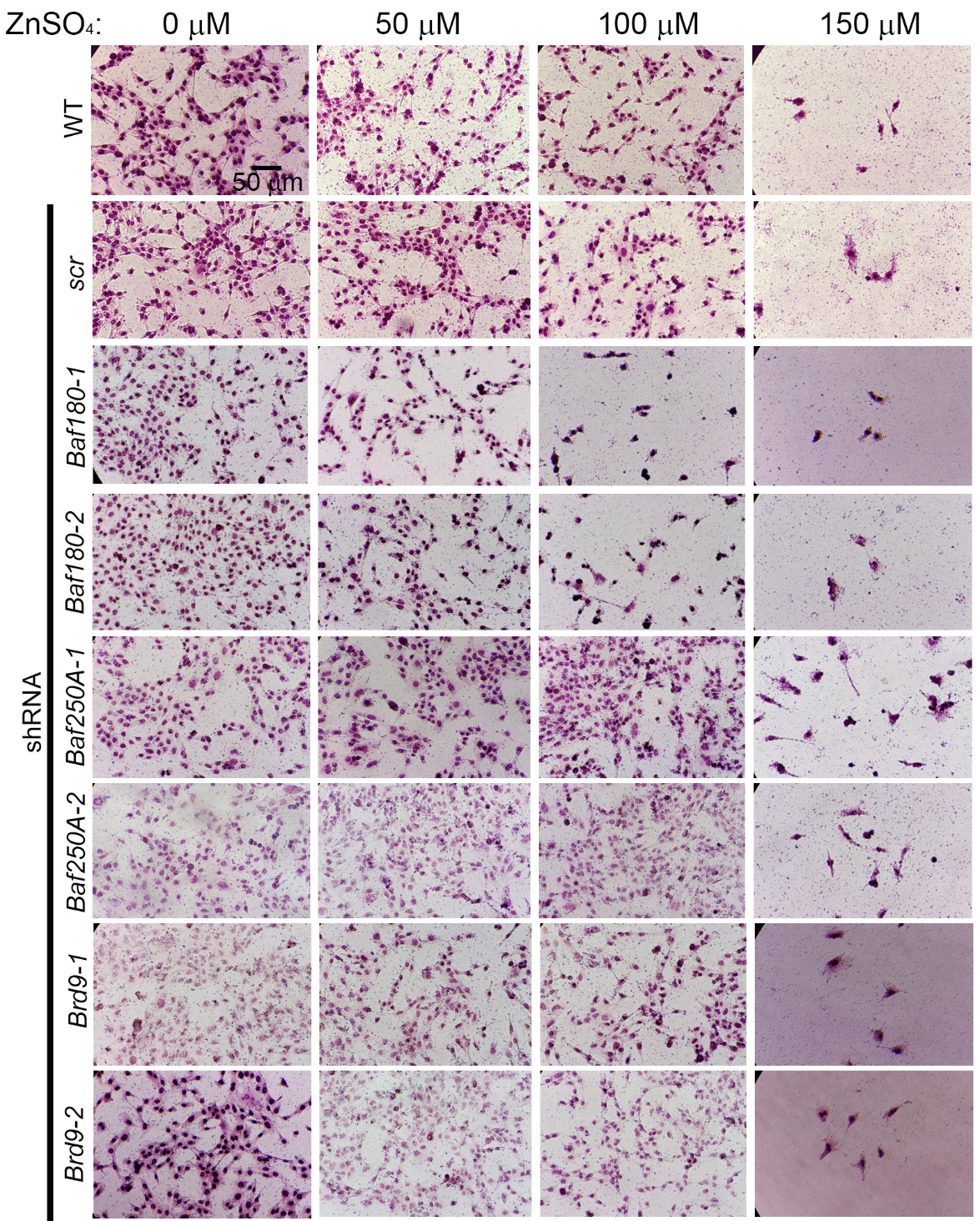


**Supplemental Figure 4. ZnSO_4_ supplementation impairs *Baf180* KD myoblast proliferation but restores the proliferation defect of *Baf250A* and *Brd9* KD myoblasts.** Representative light micrographs of proliferating WT, scr, *Baf180* KD*, Baf250A* KD*,* or *Brd9* KD C2C12 myoblasts supplemented with 0, 50, 100, and 150 μM ZnSO_4_ over 48 h and immunostained for Pax7.

**SUPPLEMENTAL FIGURE 5**

**
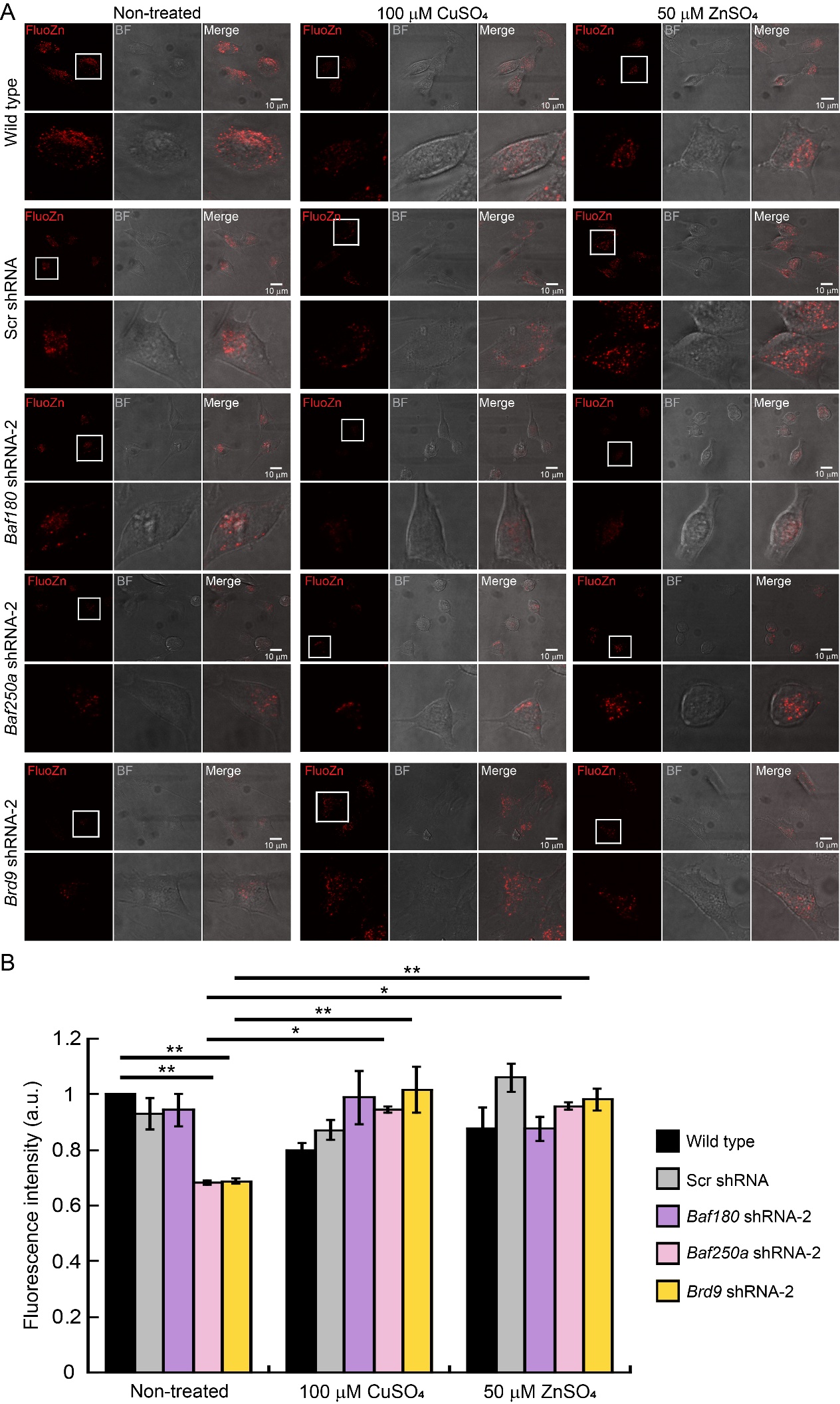
**

**Supplemental Figure 5. Distribution of labile Zn in control and SWI/SNF KD proliferating C2C12 myoblasts. (A)** Confocal microscopy live-cell analysis of labile Zn in wild-type (WT), Scr control, and KD myoblasts for *Baf180*, *Baf250a*, and *Brd9* under cultured for 48 h in basal, untreated media (NT) or supplemented with 100 μM CuSO_4_ or 50 μM ZnSO_4_. Labile Zn (red) was detected using FluoZn3. *Baf250a* and *Brd9* KD myoblasts exhibited a decreased signal of labile Zn when cells are cultured in basal media but recover upon not supplemented with metals. Scale bar: 10 μm. **(B)** Quantification of the fluorescence of live-cell imaging for Zn with the FluoZn3 from proliferating C2C12 myoblasts. N = 3, *P < 0.05; ** P < 0.01.

**SUPPLEMENTAL FIGURE 6**

**
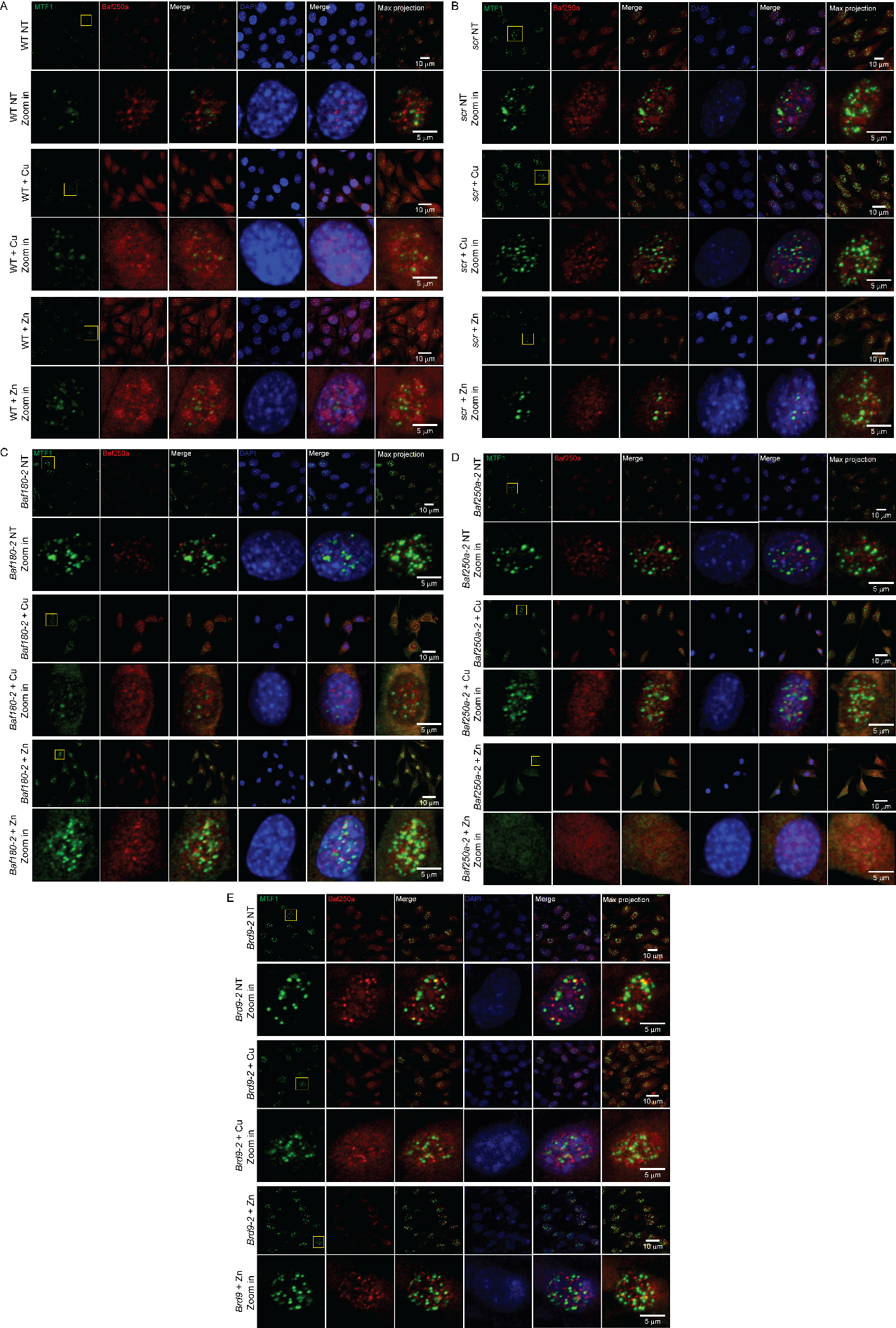
**

**Supplemental Figure 6. Expression and nuclear localization of Baf250a in proliferating C2C12 myoblasts.** Representative confocal microscopy images showing the expression and localization of Baf250a (red), MTF1 (green), and DAPI (blue) in proliferating wild type (A), scr (B), *Baf180* KD (C), *Baf250a* KD (D) and *Brd9* KD (E) C2C12 myoblasts. Nuclei are counterstained with DAPI (blue). Non-treated myoblasts exhibit nuclear Baf250a localization, with limited colocalization observed between Baf250a and MTF1 (See quantification in Fig. 4F). Each panel presents an overview of immunostained cells (upper panel, scale bar = 10 μm) and a zoomed-in view of a highlighted cell (lower panel, scale bar = 5 μm).

**SUPPLEMENTAL FIGURE 7**


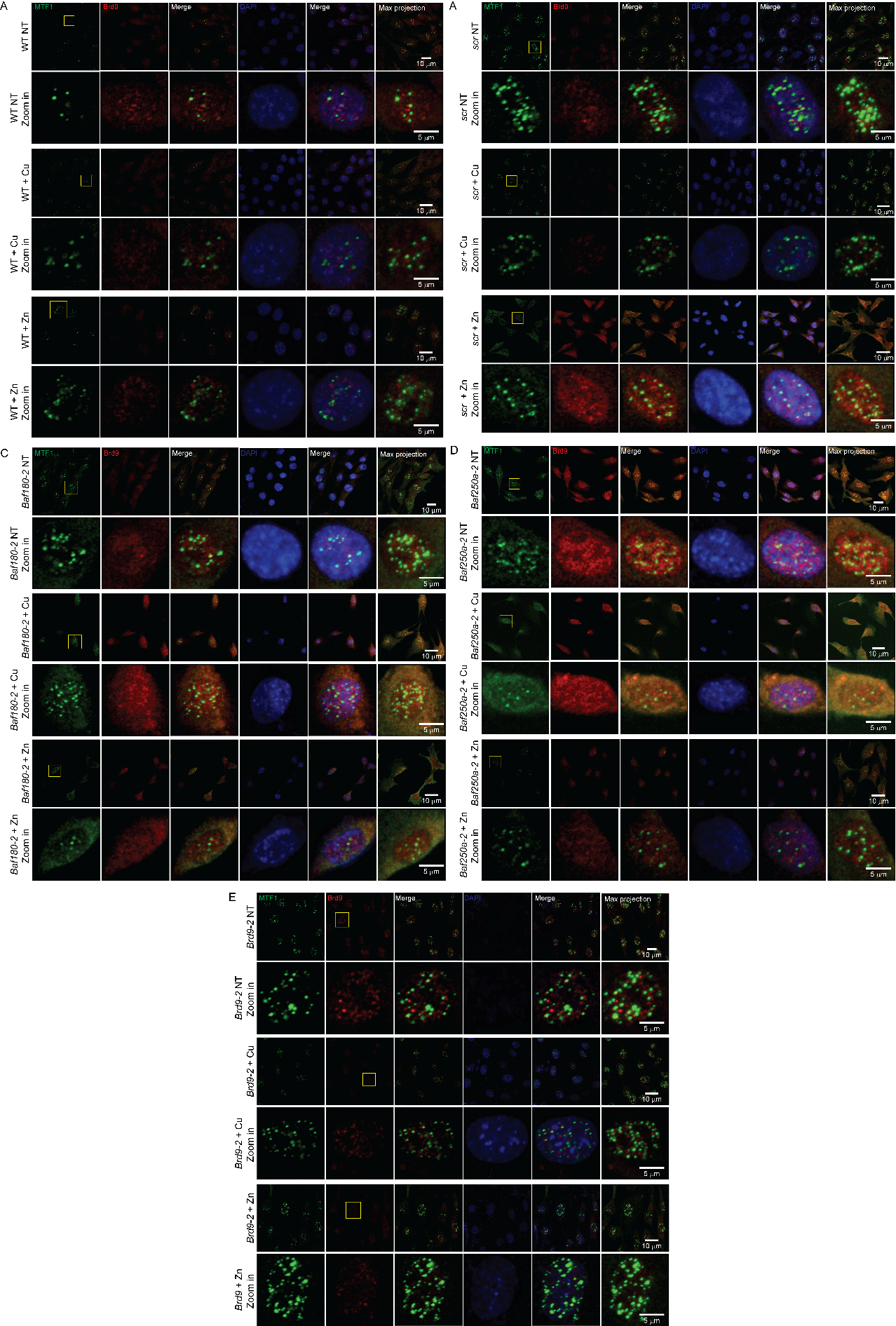


**Supplemental Figure 7. Expression and nuclear localization of Brd9 in proliferating C2C12 myoblasts.** Representative confocal microscopy images showing the expression and localization of Brd9 (red), MTF1 (green), and DAPI (blue) in proliferating wild type (A), scr (B), *Baf180* KD (C), *Baf250a* KD (D) and *Brd9* KD (E) C2C12 myoblasts. Nuclei are counterstained with DAPI (blue). Non-treated myoblasts exhibit nuclear Brd9 localization, with no colocalization observed between Brd9 and MTF1 (See quantification in Fig. 4F). Each panel presents an overview of immunostained cells (upper panel, scale bar = 10 μm) and a zoomed-in view of a highlighted cell (lower panel, scale bar = 5 μm).

**SUPPLEMENTAL FIGURE 8**

**
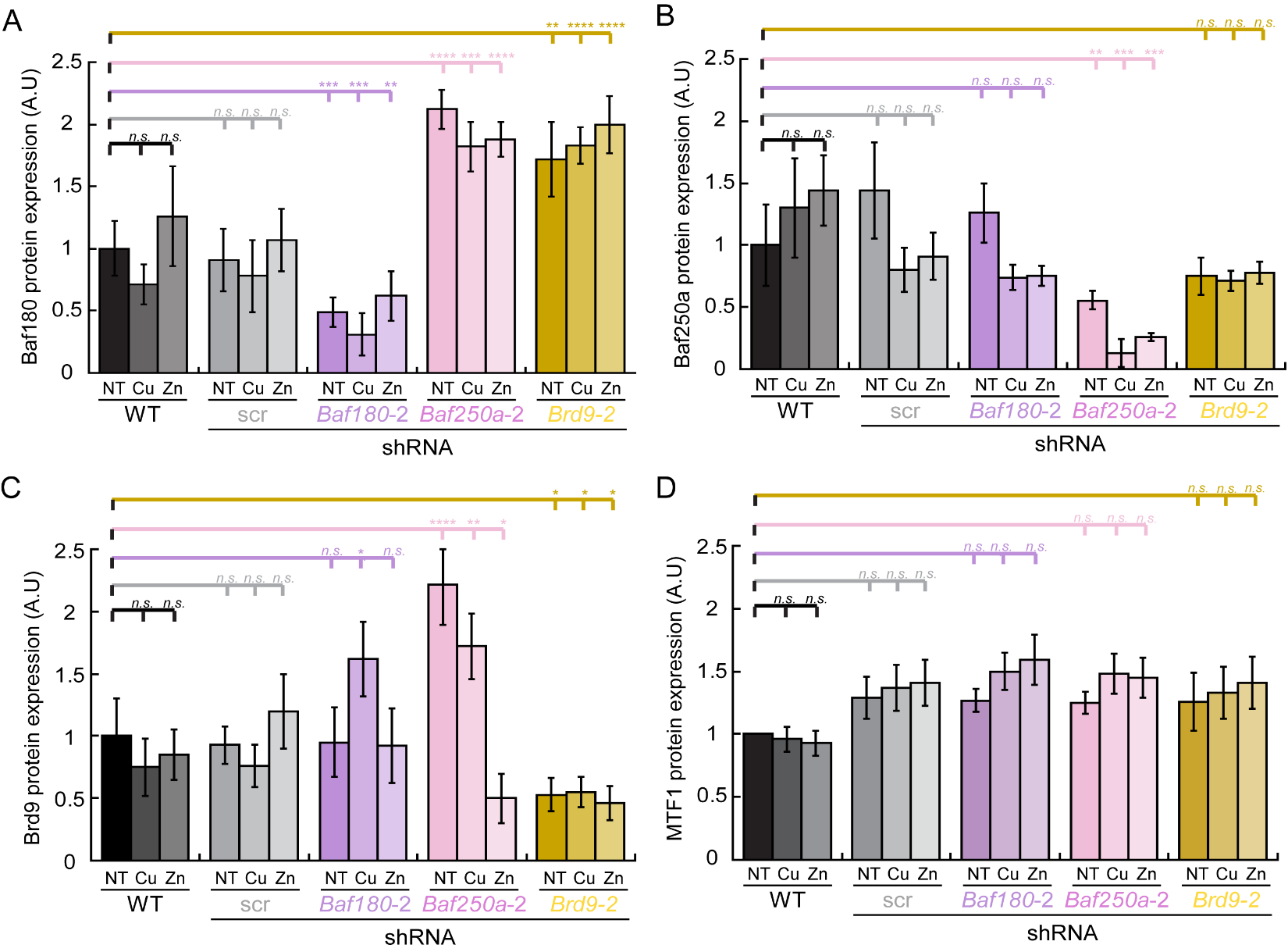
** **Supplemental Figure 8. Quantification of SWI/SNF subunit and MTF1 protein levels in proliferating C2C12 myoblasts.** Confocal microscopy-based quantification of protein expression for **(A)** Baf180, **(B)** Baf250a, **(C)** Brd9 and **(D)** MTF1 in wild type (WT), scr, *Baf180* KD, *Baf250a* KD, and *Brd9* KD myoblasts under different conditions: Untreated (NT), CuSO_4_ (100 μM, 48h) and ZnSO_4_ treatment (50 μM, 48h). Data represents the mean ± SE of three independent biological replicates. *P < 0.05, **P < 0.01, ****P < 0.0001.

**SUPPLEMENTAL FIGURE 9**­**A**
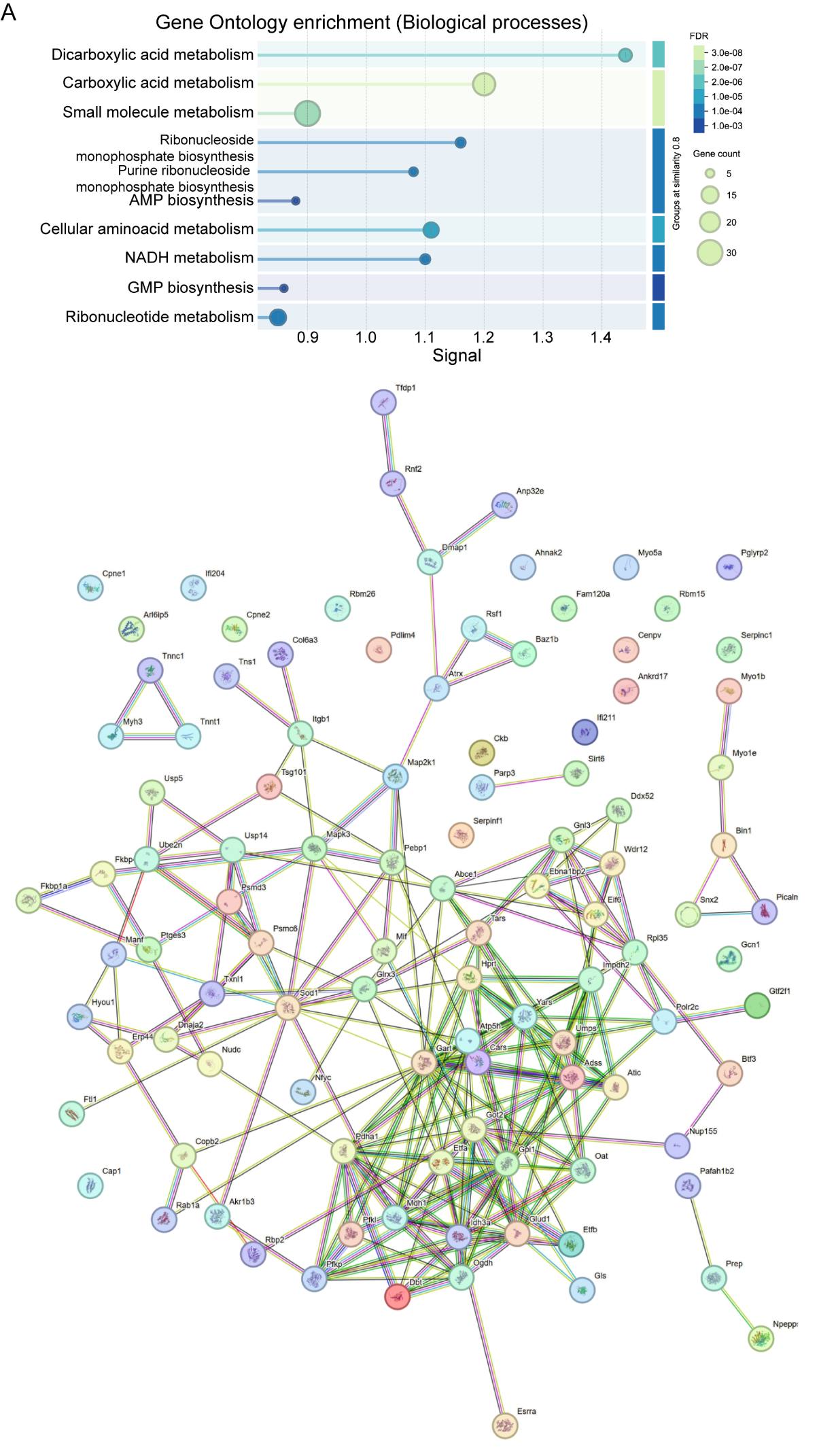
 **SUPPLEMENTAL FIGURE 9B**
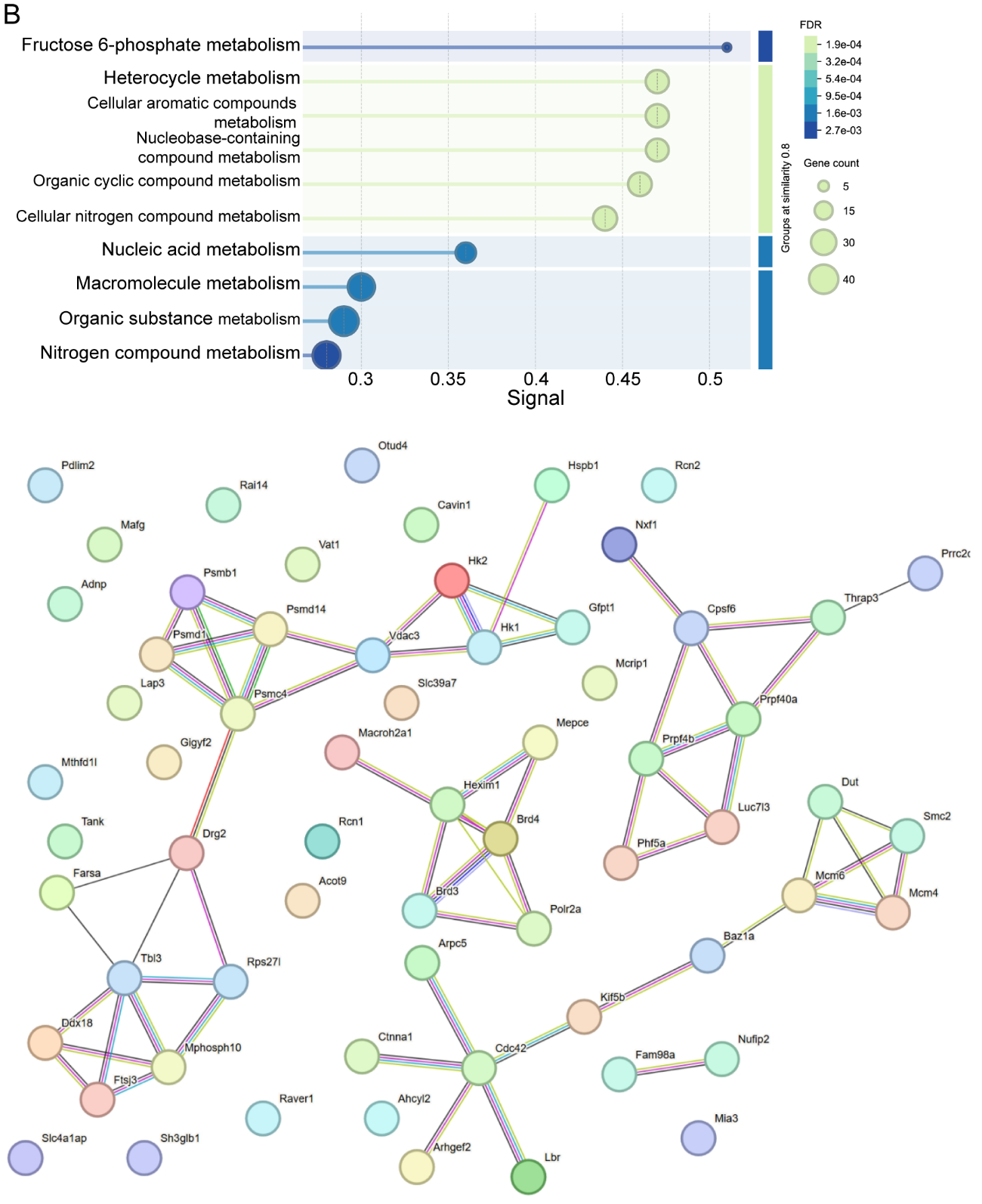
 **SUPPLEMENTAL FIGURE 9**­**C**
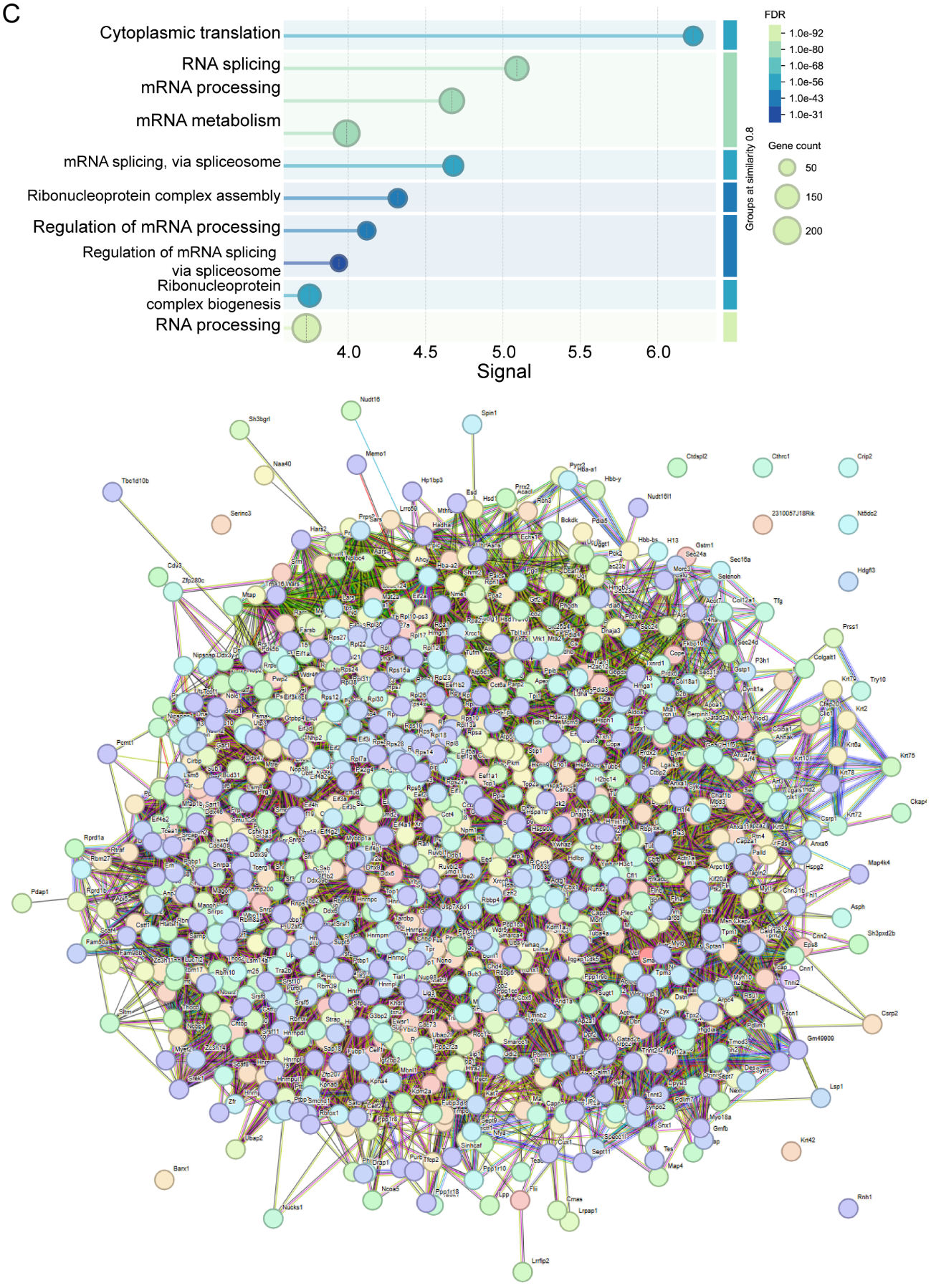


**Figure 9. Cu-dependent reorganization of the MTF1 interactome revealed by IP-mass spectrometry and PPI network analysis.** Unbiased IP-mass spectrometry of myoblasts identified MTF1-interacting proteins enriched for metabolic and biosynthetic pathways. Enriched GO categories (top) and corresponding PPI nodes identified by STRING analyses (bottom) are shown for proliferating C2C12 myoblasts cultured in the absence of Cu (-Cu; **A**) or under Cu-sufficient conditions (+Cu; **B**), and proteins common to both Cu-deficient and Cu-sufficient conditions (**C**). Note that the shared interactome included the SWI/SNF components Baf180 (PBRM1) and Baf250a (ARID1A), but not Brd9, supporting biochemical and imaging evidence of MTF1 association with chromatin-remodeling complexes.

**SUPPLEMENTAL FIGURE 10**


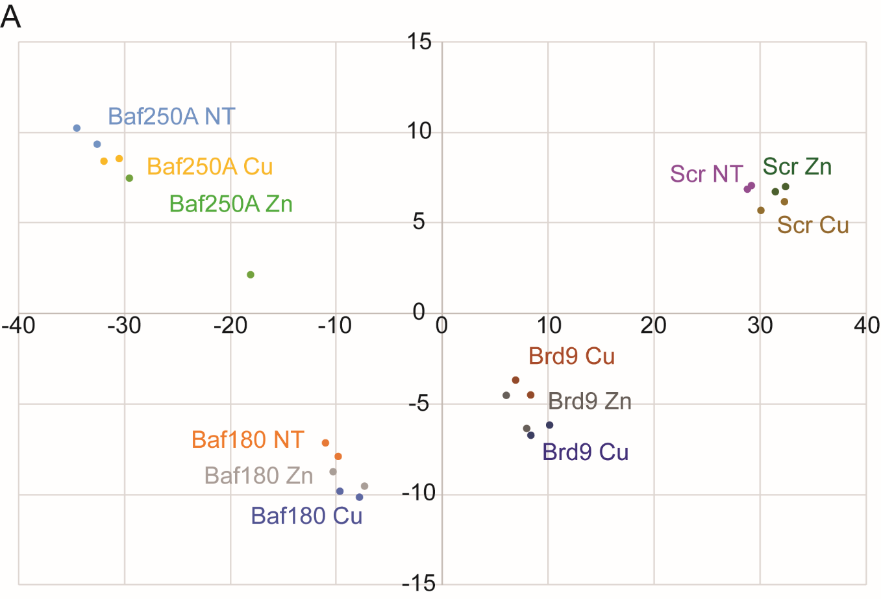


**Supplemental Figure 10. Principal component analysis reveals consistent global transcriptome profiles across conditions.** Principal Component Analysis (PCA) comparing global gene expression profiles of proliferating Scr control, and *Baf180*, *Baf250a*, and *Brd9* KD myoblasts, under both untreated conditions and following 48-hour treatment with 100 μM CuSO_4_ or 50 μM ZnSO_4_. PCA clustering indicates that transcriptomic profiles remain consistent within each knockdown condition, regardless of metal treatment.

**SUPPLEMENTAL FIGURE 11
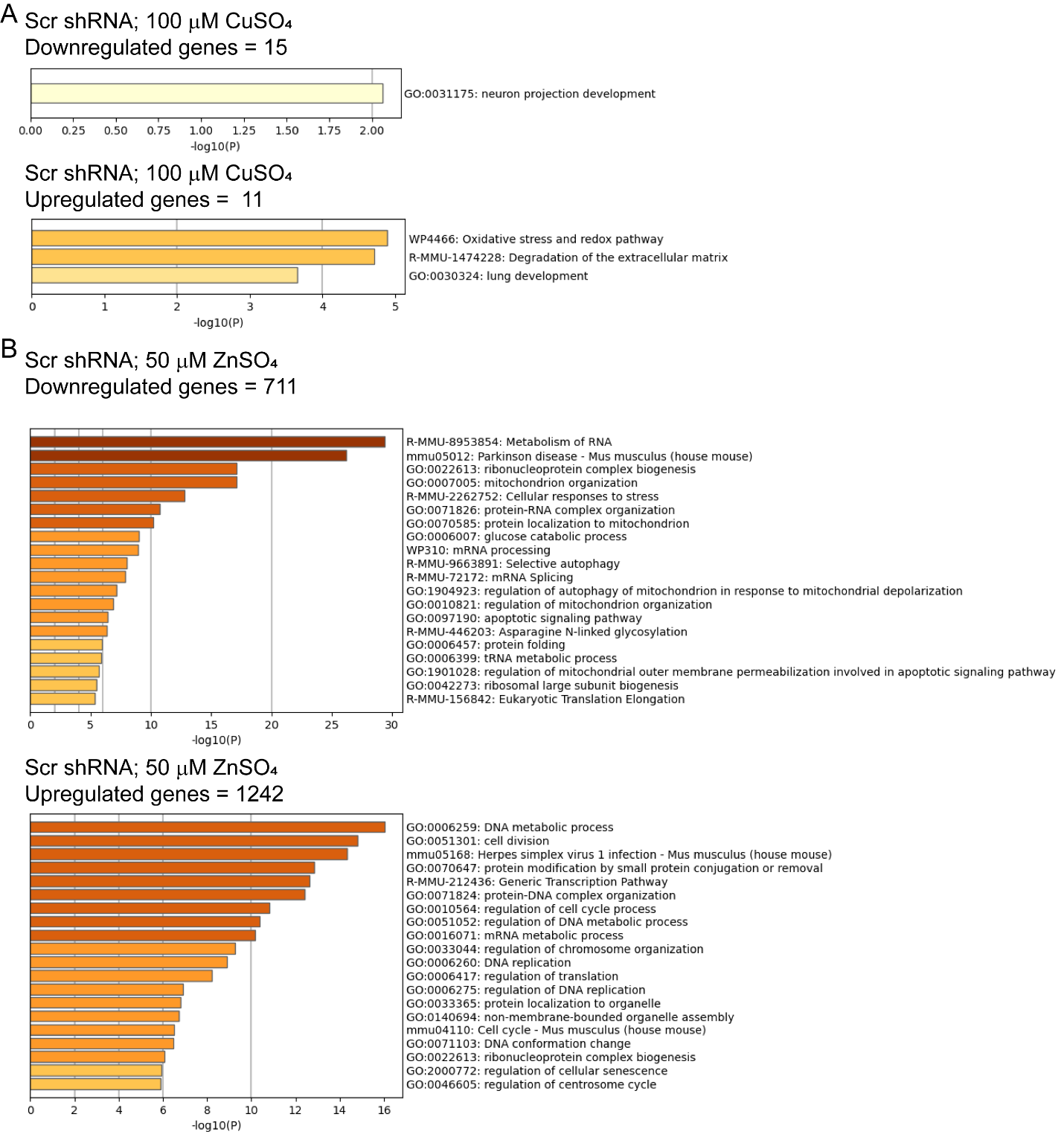
**

**Supplemental Figure 11. GO analysis of DEGs in scr control myoblasts supplemented with metals.** DEG identified from scr control cells cultured in proliferation media supplemented with 100 μM CuSO_4_ **(A)** or 50 μM ZnSO_4_ (B) were compared to the same cell line cultured in the absence of metals (basal media). The patterns represent downregulation and upregulation of DEGs shown in **Supp. Table 4**.

**SUPPLEMENTAL FIGURE 12**

**
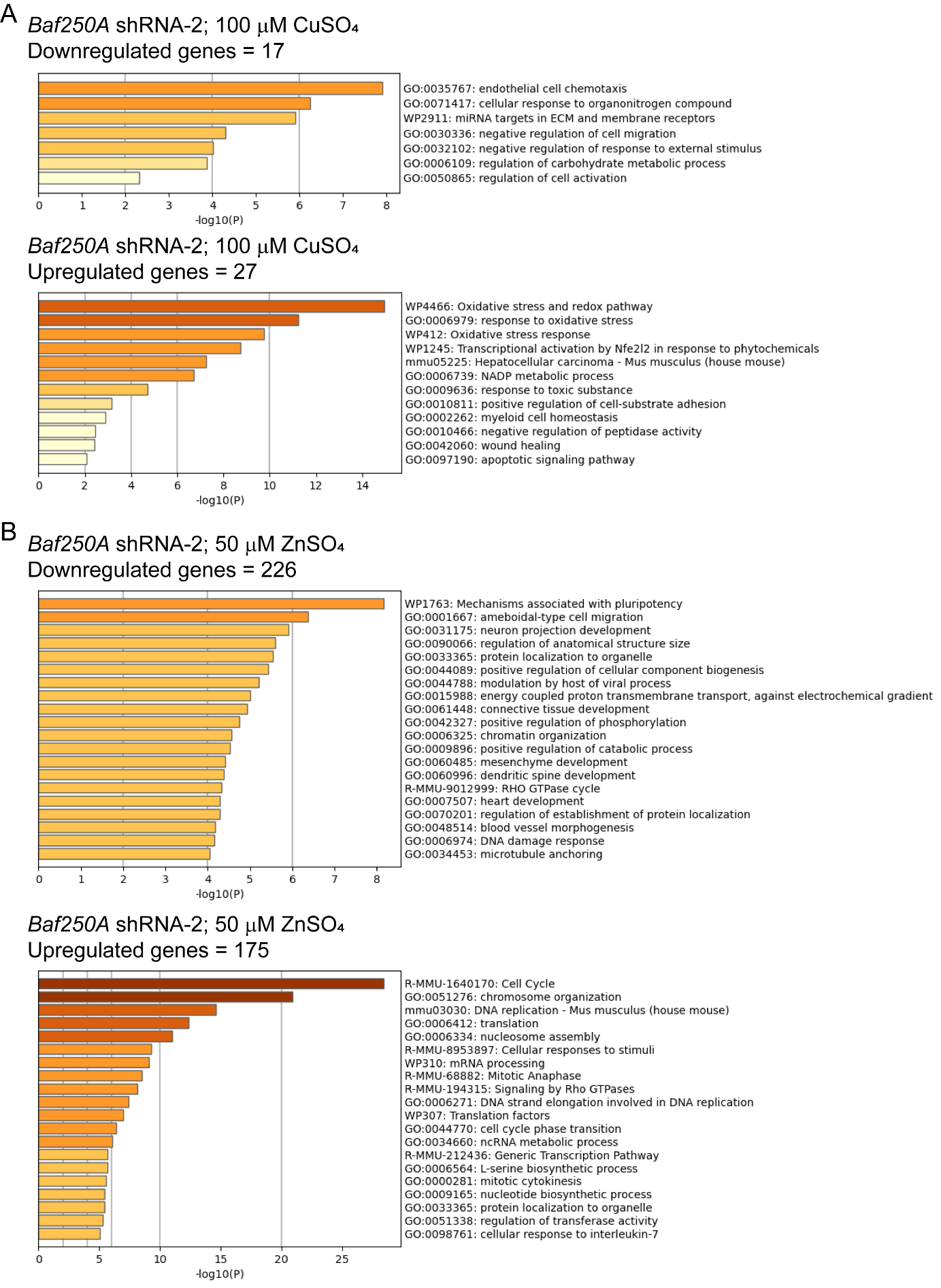
**

**Supplemental Figure 12.** GO analysis of DEGs in *Baf250a* KD myoblasts supplemented with metals. DEG identified from *Baf250a* KD cells cultured in proliferation media supplemented with 100 μM CuSO_4_ **(A)** or 50 μM ZnSO_4_ **(B)** were compared to the same cell line cultured in the absence of metals (basal media). The patterns represent downregulation and upregulation of DEGs shown in **Supp. Table 4**.

**SUPPLEMENTAL FIGURE 13**

**
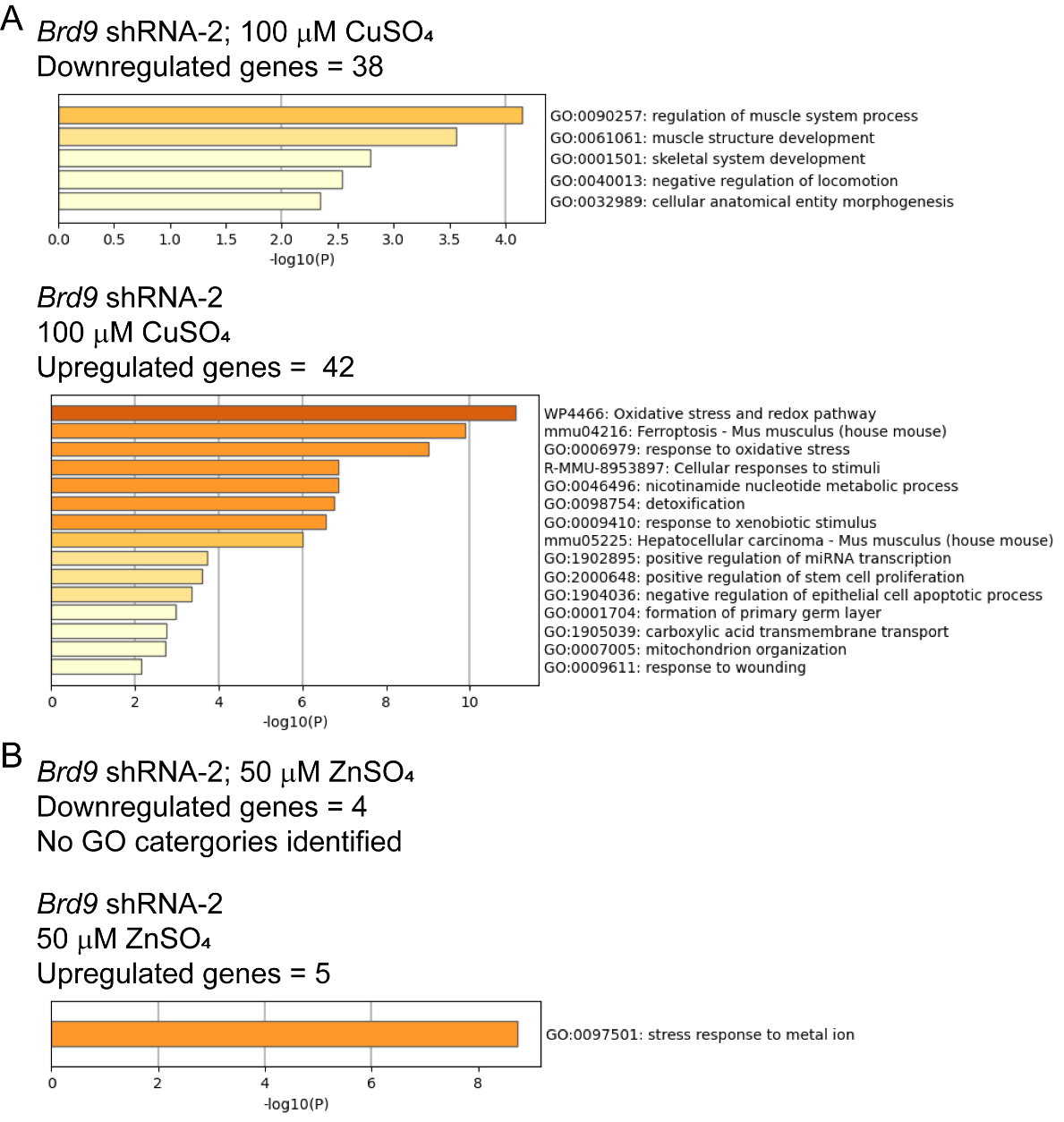
**

**Supplemental Figure 13.** GO analysis of DEGs in *Brd9* KD myoblasts supplemented with metals. DEG identified from *Brd9* KD cells cultured in proliferation media supplemented with 100 μM CuSO_4_ **(A)** or 50 μM ZnSO_4_ (B) were compared to the same cell line cultured in the absence of metals (basal media). The patterns represent downregulation and upregulation of DEGs shown in **Supp. Table 4**.

**SUPPLEMENTAL FIGURE 14**


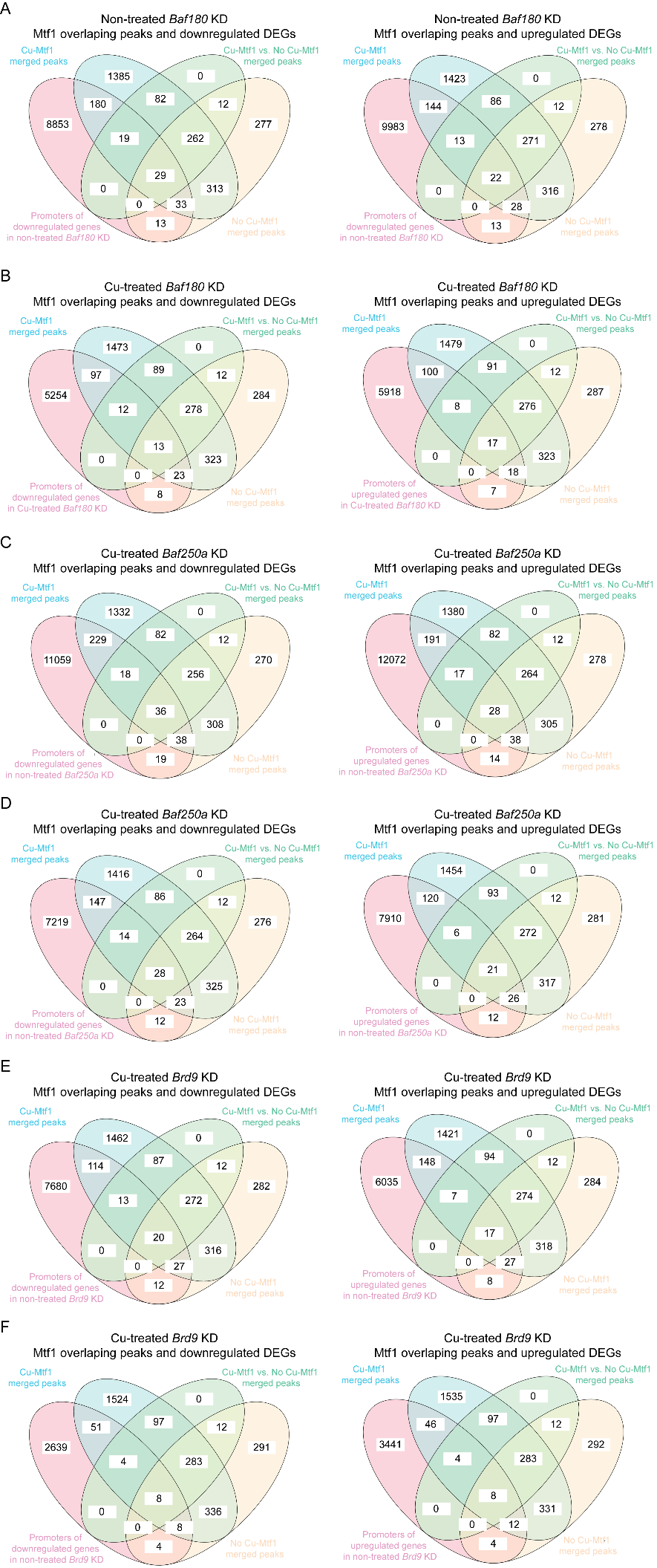


**Supplemental Figure 14. MTF1 chromatin binding correlates with DEGs in SWI/SNF KD myoblasts under Cu exposure.** The Venn diagrams illustrate the overlap of differentially expressed genes (DEGs) that are either upregulated or downregulated across *Baf180* **(A, B)**, *Baf250a* **(C, D)**, and *Brd9* **(E, F)** KD myoblasts, cultured with or without Cu treatment. These transcriptional changes were further integrated with MTF1 chromatin occupancy data obtained from CUT&RUN analysis of non-treated and Cu-treated cells, providing insight into the regulatory landscape influenced by MTF1. Notably, a subset of DEGs across the different SWI/SNF subunit KD conditions overlapped with MTF1 binding sites, suggesting that MTF1 directly regulates a fraction of genes affected by SWI/SNF disruption. The shared and unique gene sets highlight the interplay between SWI/SNF chromatin remodelers and MTF1 in orchestrating transcriptional responses to Cu, reinforcing the role of these factors in metal-responsive gene regulation and myoblast adaptation to metal stress.

**SUPPLEMENTAL TABLES**

**Supplemental Table 1. Sequences of shRNA used in this study (2, 3).**

| *Baf250A* shRNA1 | CCGGCTTTATAGTATGGCGAGTTAACTCGAGTTAACTCGCCATACTATAAAGTTTTTG | TRCN0000238304 |
| --- | --- | --- |
| *Baf250A* shRNA2 | CCGGCCTAGGCAGCCTAACTATAATCTCGAGATTATAGTTAGGCTGCCTAGGTTTTTG | TRCN0000238306 |
| *Brd9* shRNA1 | CCGGTGGACTTTGGCACGATGAAAGCTCGAGCTTTCATCGTGCCAAAGTCCATTTTTG | TRCN0000225737 |
| *Brd9* shRNA2 | CCGGCACCGAATGGTGTCCAATAAGCTCGAGCTTATTGGACACCATTCGGTGTTTTTG | TRCN0000225739 |
| *Baf180* shRNA1 | CCGGTGTGAAGTTGGTCCTAGTTTACTCGAGTAAACTAGGACCAACTTCACATTTTTG | TRCN0000304680 |
| *Baf180* shRNA2 | CCGGGTGCAATATCCAGACTATTATCTCGAGATAATAGTCTGGATATTGCACTTTTTG | TRCN0000304681 |
| scr shRNA | CCGGCAACAAGATGAAGAGCACCAACTCGAGTTGGTGCTCTTCATCTTGTTGTTTTT | pLKO.1-puro non-target shRNA control plasmid DNA MFCD07785395 SHC002 |

**Supplemental Table 4. Summary of DEG genes from metal-treated KD and scr control cells.** Data shows the differences within each strain upon metal supplementation


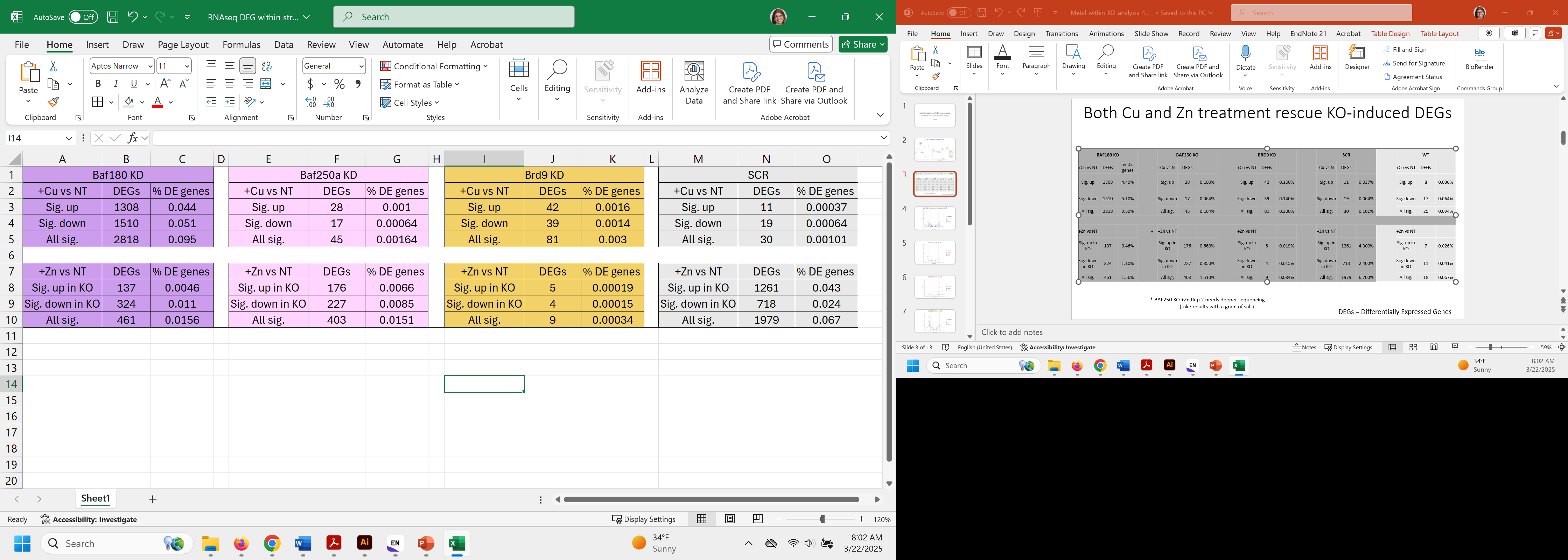


**SUPPLEMENTAL REFERENCES**

1. Paskavitz, A. L., Quintana, J., Cangussu, D., Tavera-Montanez, C., Xiao, Y., Ortiz-Miranda, S., Navea, J. G., and Padilla-Benavides, T. (2018) Differential expression of zinc transporters accompanies the differentiation of C2C12 myoblasts. *J Trace Elem Med Biol* **49**, 27-34

2. Padilla-Benavides, T., Olea-Flores, M., Sharma, T., Syed S.A., Witwicka H., Zuñiga-Eulogio, M., Zhang, K., Navarro-Tito, N. and Imbalzano A.N. . (2023) Differential Contributions of mSWI/SNF Chromatin Remodeler Sub-Families to Myoblast Differentiation. *International Journal of Molecular Sciences* **24**, 11256

3. Padilla-Benavides, T., Olea-Flores, M., Nshanji, Y., Maung, M.T., Syed, S.A. and Imbalzano, A.N. (2022) Differential requirements for different subfamilies of the mammalian SWI/SNF chromatin remodeling enzymes in myoblast cell cycle progression and expression of the Pax7 regulator. *Biochimica et Biophysica Acta (BBA) - Gene Regulatory Mechanisms*
